# NOX2‐Driven Oxidative Stress Promotes EndMT and Uncouples Angiogenesis–Osteogenesis at the Bone–Implant Interface in Diabetes

**DOI:** 10.1002/advs.202517885

**Published:** 2025-11-10

**Authors:** Zimei Wu, Qiaodan Hou, Yang Liu, Tingting Chen, Kunkun Yang, Luyao Li, Lin Wang

**Affiliations:** ^1^ Department of Biochemistry, SUSTech Homeostatic Medicine Institute School of Medicine Southern University of Science and Technology Shenzhen Guangdong 518055 P. R. China; ^2^ Emergency and Disaster Medical Center The Seventh Affiliated Hospital Sun Yat‐Sen University Shenzhen 518107 P. R. China; ^3^ Department of Biomedical Engineering Southern University of Science and Technology Nanshan District Shenzhen 518055 P. R. China

**Keywords:** angiogenesis–osteogenesis coupling, diabetes, EndMT, NOX2, osseointegration, titanium–bone interface

## Abstract

Diabetes undermines implant integration, yet how the bone–implant interface (BII) can be rationally re‐engineered remains unclear. Here endothelial NOX2–driven oxidative stress is identified as a mechanistic switch that triggers endothelial‐to‐mesenchymal transition (EndMT), depletes type‐H (CD31^+^EMCN^+^) vessels, and uncouples angiogenesis from osteogenesis. In a diabetic titanium‐implant model, type‐H endothelium and adjacent osteoprogenitors declined early (≈40% loss at 2 weeks), and peri‐implant bone volume is reduced at 8 weeks (BV/TV 40.98 ± 3.96%). Pharmacologic NOX2 inhibition or endothelial‐specific Nox2 deletion restored endothelial identity, suppressed EndMT and apoptosis, rebuilt type‐H networks, and improved bone formation (BV/TV 53.15 ± 4.97%), yielding higher BV/TV and histological indices. In vitro, on titanium surfaces, NOX2 blockade rescued endothelial proliferation, migration, and adhesion architecture and re‐enabled osteogenesis in EC–OB co‐culture. Bulk RNA‐seq demonstrated pathway reversals (TGF‐β, NF‐κB/MAPK, Wnt/Notch, TNF) and attenuated EC to OB ephrin/plexin edges, consistent with guidance/adhesion rewiring. These findings position NOX2 as an actionable vascular target at the BII and suggest interface‐focused delivery (drug coatings, anti‐EndMT functional surfaces, responsive hydrogels) to re‐establish vessel–bone coupling and strengthen osseointegration in diabetes.

## Graphical abstract

NOX2‐driven oxidative stress disrupts angiogenesis–osteogenesis coupling at the diabetic bone–implant interface. Pharmacologic inhibition and endothelial‐specific NOX2 knockout restore type‐H endothelium, suppress EndMT, and improve osseointegration. Transcriptomics implicate TGF‐β, NF‐κB, MAPK, TNF, and Wnt/Notch pathways, highlighting NOX2 as a druggable node for localized, interface‐targeted therapies in diabetic bone regeneration with clinical promise.

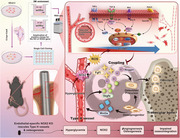

## 1 Introduction

Intraosseous titanium (Ti) implants are widely used to restore skeletal function, and long‐term success critically depends on stable osseointegration—direct bone‐implant contact at the bone‐implant interface (BII). However, in patients with type 2 diabetes mellitus (T2DM), the failure rate of implants is markedly higher—≈10–20% versus 1–3% in non‐diabetic cohorts, primarily due to impaired bone regeneration, fibrotic encapsulation, and chronic inflammation at the BII.^[^ ^1^, ^2^, ^3^ ^]^ Given the rising global prevalence of diabetes—projected to exceed 783 million people by 2045^[^ ^4^, ^5^ ^]^—mitigating implant‐related complications in T2DM is a pressing clinical priority, a burden that spans both dental and orthopedic indications, although most of the present studies focus on intraosseous Ti implants in appendicular bone.

Conventional strategies—such as systemic glycemic control or anti‐osteoporotic regimens—provide partial benefits yet rarely re‐establish the local microenvironmental homeostasis required for durable anchorage at the BII.^[^ ^6^ ^]^ Accordingly, recent efforts have shifted toward vascular‐niche modulation to re‐establish coupling between angiogenesis–osteogenesis, an essential prerequisite for bone healing and implant integration.^[^ ^7^ ^]^ A pivotal component of coupling is type H vascular endothelium (CD31^+^EMCN^+^),^[^ ^8^ ^]^ which not only support perfusion but also instruct osteoprogenitor recruitment and differentiation, thereby coordinating bone formation.^[^ ^9^, ^10^, ^11^, ^12^ ^]^ Although diabetes has been reported associated with reduced type H vessel density and a deteriorated osteogenic niche;^[^ ^13^, ^14^ ^]^ nevertheless, the mechanistic link between hyperglycemic vascular injury and endothelial‐osteogenic crosstalk at the BII remains unclear.

At the molecular level, chronic hyperglycemia activates endothelial NADPH oxidase 2 (NOX2), a major source of reactive oxygen species (ROS), thereby precipitating oxidative stress and endothelial dysfunction.^[^ ^13^, ^14^, ^15^, ^16^, ^17^, ^18^ ^]^ Furthermore, ROS can promote endothelial‐mesenchymal transition (EndMT), a phenotypic shift that remodels the extracellular matrix (ECM) and impairs vascular functions relevant to bone repair.^[^ ^19^, ^20^, ^21^ ^]^ These observations led us to hypothesize that NOX2‐driven ROS accumulation induces EndMT, resulting in loss of type H vascular endothelium and uncoupling of angiogenesis from osteogenesis at the BII under diabetic conditions. Unlike prior studies that primarily focused on optimizing implant surface properties^[^ ^22^, ^23^, ^24^ ^]^ or relied on systemic metabolic control,^[^ ^25^, ^26^, ^27^, ^28^ ^]^ our approach targets endothelial NOX2 at the diabetic BII and integrates pharmacological inhibition with endothelial‐specific knockout, coupled with multimodal readouts.

Here, we combined pharmacologic inhibition and an endothelial‐specific NOX2 conditional knockout (EC‐NOX2^−/−^) with micro‐CT, histology, immunofluorescence, flow cytometry, and transcriptomic profiling to rigorously test this hypothesis in Ti‐implanted murine diabetic models. Our study reveals that targeting NOX2 restores type H endothelium, suppresses EndMT markers, and improves peri‐implant bone formation, offering a novel approach to enhancing osseointegration in diabetes. Transcriptome profiling revealed reversal of ECM‐remodeling programs and attenuation of EndMT‐linked signaling pathways, providing new mechanistic insights into NOX2‐driven endothelial dysfunction in diabetic bone healing. These findings underscore the potential of NOX2 as a therapeutic target for improving diabetic implant outcomes, although the conclusions are confined to murine T2DM models and Ti implants within the studied time window, as well as the specific delivery routes and dosing regimens used in mice, warranting further evaluation across skeletal sites and delivery formats.

## 2 Results

### 2.1 Diabetes Compromises Osseointegration and Precipitates Early Angiogenesis–Osteogenesis Uncoupling at the BII

**Figure 1:**
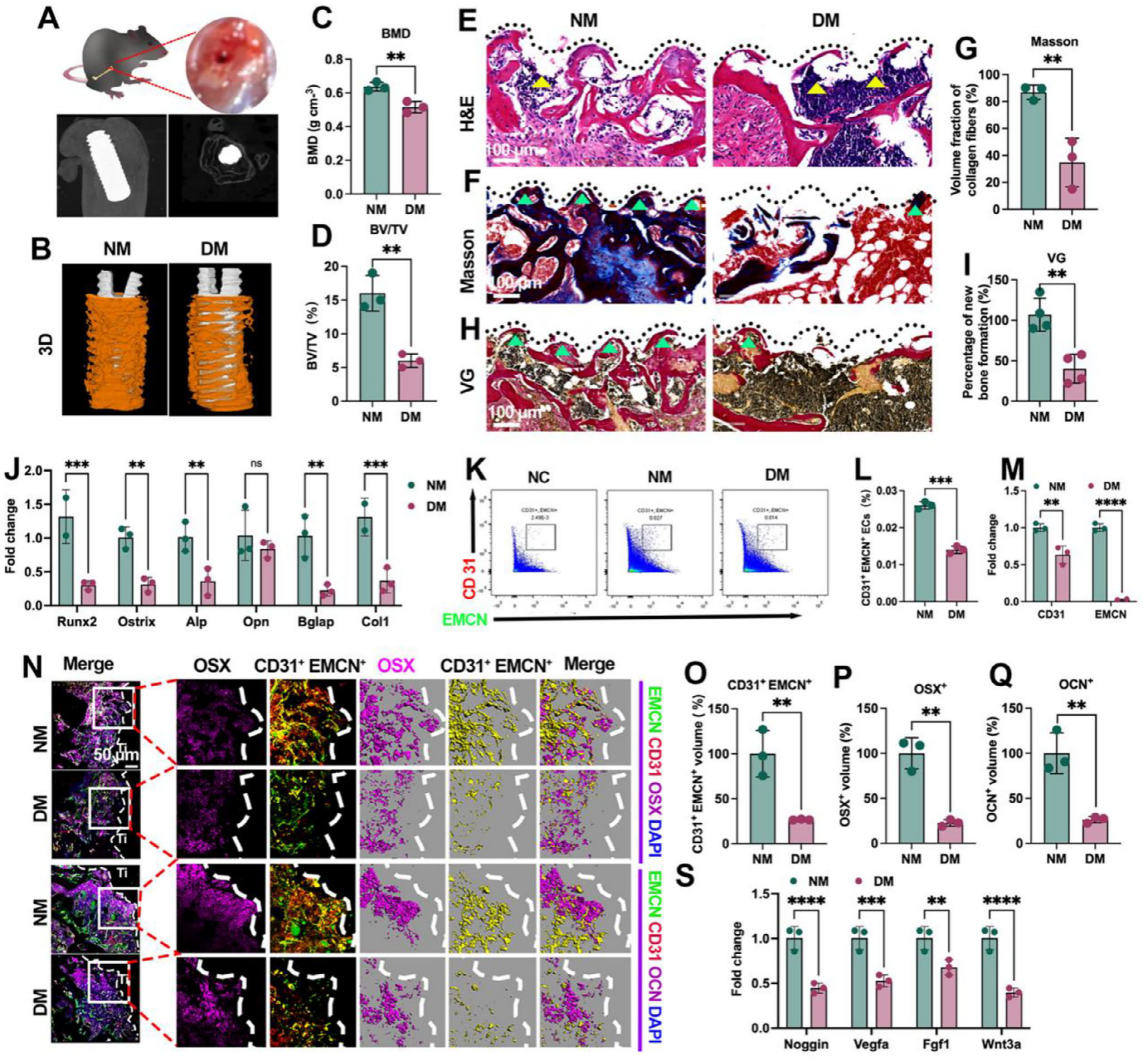
Diabetes impairs osseointegration and induces early angiogenesis–osteogenesis uncoupling at the BII. A) Ti screw placement and radiographic verification. B) micro‐CT 3D reconstructions with peri‐implant ROI (implant, white; new bone, yellow). C,D) BMD and BV/TV within the ROI. E) H&E sections showing peri‐implant bone formation (dashed line, implant–tissue interface). F,G) Masson's trichrome (collagen‐rich mineralized bone, blue) and histomorphometric quantification. H,I) Van Gieson (new bone, red) and histomorphometric quantification. J) qPCR of osteogenic genes (Runx2, Osterix, Alp, Opn, Bglap, Col1a1). K) Flow‐cytometry gating for type‐H (CD31⁺EMCN⁺) versus total ECs; L) proportion of CD31⁺EMCN⁺ ECs. M) qPCR for type‐H markers (CD31, EMCN). N) Two‐week BII immunofluorescence: CD31 (red), EMCN (green), type‐H (yellow pseudocolor), OSX or OCN (purple), DAPI (blue); maximum‐intensity projections and surface renderings. O,P) Quantification of type‐H endothelium and OSX⁺ osteoprogenitors at 2 weeks. Q) Quantification of OCN⁺ cells and type‐H endothelium at 8 weeks. S) qPCR of coupling factors (Noggin, Vegfa, Fgf1, and Wnt3a). Scale bars: 50 µm (N); 100 µm (E, F, H). Data presentation: mean ± SD; *n* = 3 per group. Statistics: two‐tailed Student's t‐tests for two‐group comparisons and one‐way ANOVA with Tukey's post hoc test for ≥3 groups, as appropriate depending on normality; *P* < 0.05 (*), *P* < 0.01 (**), *P* < 0.001 (***), *P* < 0.0001 (****); ns, not significant.

Ti screws were placed into femoral defects and positioning was verified radiographically (**Figure** **1**A). At 8 weeks, micro‐CT 3D reconstructions showed reduced peri‐implant bone in diabetic mice (DM) compared with non‐diabetic controls (NM) (Figure 1B), with significantly lower bone mineral density (BMD) and bone volume fraction (BV/TV) within the peri‐implant ROI (Figure 1C,D), indicating compromised osseointegration. Histology corroborated these findings: H&E sections revealed fibrous tissue replacing the bone–implant interface in DM versus compact bone in NM (Figure 1E). Masson's trichrome and Van Gieson (VG) staining demonstrated markedly reduced collagen‐rich, mineralized new bone in DM (Figure 1F,H), with corresponding decreases in collagen fraction and new‐bone area on histomorphometry (Figure 1G,I). Consistently, qPCR of peri‐implant tissue showed down‐regulation of osteogenic markers—Runx2, Osterix, Alp, Bglap, Col1a1—with Opn unchanged (Figure 1J). These data establish an endpoint impairment of peri‐implant osteogenesis under diabetic conditions.

To probe early coupling at the interface, we quantified endothelial subsets by flow cytometry using a standardized gating strategy for type‐H endothelium (CD31⁺EMCN⁺) versus total ECs (Figure S1, Supporting Information). DM bone displayed a lower fraction of CD31⁺EMCN⁺ endothelial cells (Figure 1K,L), and CD31**/**EMCN transcripts were reduced (Figure 1M). At 2 weeks, whole‐mount immunofluorescence revealed OSX⁺ osteoprogenitors (purple) arranged around type‐H vessels (CD31, red; EMCN, green) in NM, whereas both components were diminished in DM (Figure 1N). Quantitatively, type‐H endothelial volume and OSX⁺ cell volume were reduced by 39.71 ± 5.43% and 26.66 ± 0.51%, respectively (Figure 1O,P). By 8 weeks, OCN⁺ osteoblasts and type‐H endothelium remained decreased (−26.32 ± 3.45% and −22.66 ± 3.68%, respectively; Figure 1Q), indicating a sustained coupling deficit. Moreover, coupling factors Noggin, Vegfa, Fgf1, and Wnt3a were down‐regulated in DM peri‐implant tissue (Figure 1S), suggesting a microenvironment less permissive for osteogenesis. Together, these results show that diabetes both impairs osseointegration and precipitates early angiogenesis–osteogenesis uncoupling at the BII.

### 2.2 NOX2‐Driven Oxidative Stress Underlies Endothelial Injury at the BII in Diabetes

**Figure 2:**
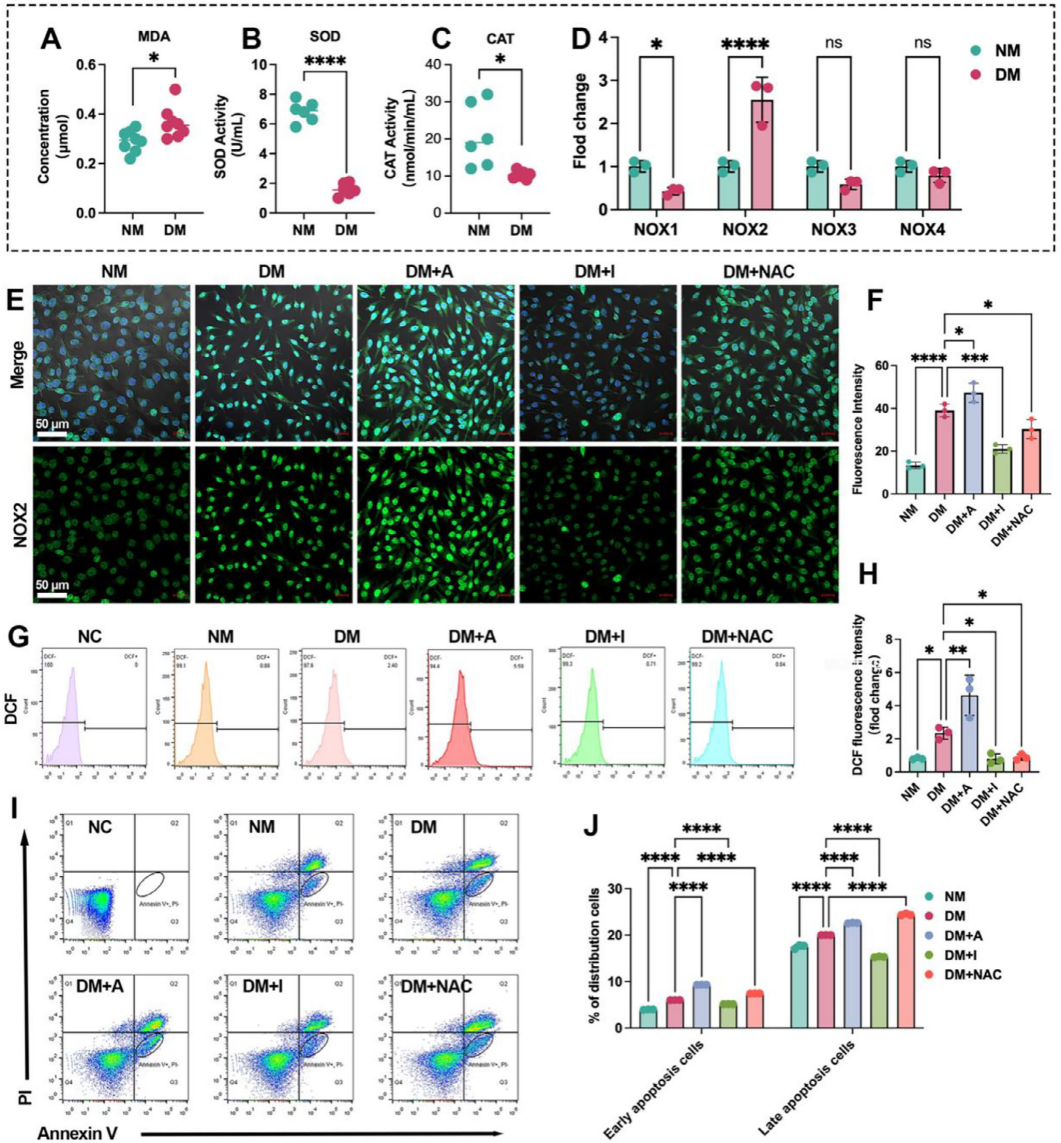
NOX2 upregulation and ROS accumulation associate with endothelial injury at the BII in diabetes. A–C) Oxidative stress in peri‐implant bone: MDA concentration and activities of SOD and CAT. D) qPCR of Nox1–4 in peri‐implant tissue. E) Immunofluorescence of NOX2 in bEnd.3 cultured on Ti under NM, DM, DM+A (TMF, NOX2 agonist), DM+I (GSK2795039, NOX2 inhibitor), and DM+NAC (ROS scavenger). F) Quantification of NOX2 fluorescence. G,H) Intracellular ROS by DCF flow cytometry and quantification. I,J) Annexin V/PI flow cytometry and quantification of early/late apoptosis under the same treatments. Scale bars:50 µm. Data presentation: mean ± SD; *n* = as indicated in panels. Statistics: two‐tailed Student's t‐tests for two‐group comparisons and one‐way ANOVA with Tukey's post hoc test for ≥ 3 groups, as appropriate depending on normality; *P* < 0.05 (*), *P* < 0.01 (**), *P* < 0.001 (***), *P* < 0.0001 (****); ns, not significant.

To explore the mechanistic link between diabetes‐induced oxidative stress and impaired angiogenesis at the BII, we first assessed peri‐implant oxidative stress markers in bone tissue surround Ti implant. The level of malondialdehyde (MDA), a lipid peroxidation by‐product, was elevated in DM versus NM (0.37 ± 0.06 vs 0.29 ± 0.04, *p* < 0.05), while antioxidant enzyme activities, including superoxide dismutase (SOD) and catalase (CAT), were reduced (**Figure** **2**A–C). These results indicate a milieu with compromised defenses at the interface. Given that NADPH oxidases (NOX) are major sources of ROS in endothelial cells,^[^ ^29^, ^30^ ^]^ we profiled NOX1‐4 isoforms by qPCR in peri‐implant tissues. NOX2 expression was upregulated by ≈1.5‐fold in DM relative to NM (Figure 2D), whereas NOX1, NOX3, and NOX4 showed no significant changes.

To further clarify the role of NOX2 in diabetes‐associated endothelial dysfunction, we evaluated how pharmacological modulation of NOX2 alters intracellular ROS and apoptosis in endothelial cells (ECs) cultured on Ti surfaces under diabetic conditions. Immunofluorescence confirmed NOX2 upregulation in ECs in DM media (Figure 2E), and quantification (Figure 2F) showed that a NOX2 agonist 4′,6,7‐trimethoxyisoflavone (TMF; DM+A), increased NOX2 signal,^[^ ^31^ ^]^ whereas the NOX2 inhibitor GSK2795039 (an inhibitor of NOX2,^[^ ^32^ ^]^ DM+I) suppressed it. Consistently, intracellular ROS measured by DCF fluorescence was lowered by NOX2 inhibition (DM+I) compared with DM (Figure 2G–H). Consistent with the DCF cytometry, live‐cell DCF imaging of bEnd.3 on Ti revealed low basal ROS in NM, a diffuse increase under DM, and a marked further elevation with NOX2 activation (DM+A), whereas NOX2 inhibition (DM+I) or ROS scavenging (DM+NAC) reduced the signal toward NM levels (Figure S2, Supporting Information). Consistent with an endothelial origin of oxidative stress, MC3T3 osteoblasts cultured alone on Ti showed no detectable change in DCF fluorescence across NM, DM, DM+A (TMF), DM+I (GSK2795039) or DM+NAC (Figure S3, Supporting Information). Thus, in our system, the diabetic medium or NOX2 modulators do not elicit a direct ROS response in osteoblasts, reinforcing that endothelial NOX2 is the principal driver of redox imbalance at the BII. Apoptotic cell death was assessed by Annexin V‐FITC/PI staining and quantified by flow cytometry (Figure 2I,J): NOX2 activation increased early/late apoptosis (9.13 ± 0.03%/ 22.60 ± 0.10%) relative to DM (6.03 ± 0.02% /20.01 ± 0.02%, respectively). whereas NOX2 inhibition reduced apoptosis (5.14 ± 0.01%/15.31 ± 0.08%). Collectively, these data implicate NOX2 upregulation in driving oxidative stress and endothelial apoptosis at the BII in diabetes, while pharmacologic NOX2 blockade mitigates ROS accumulation and cell death.

### 2.3 Pharmacologic NOX2 Modulation Bidirectionally Tunes Endothelial Function and Osteogenesis on Titanium In Vitro

**Figure 3:**
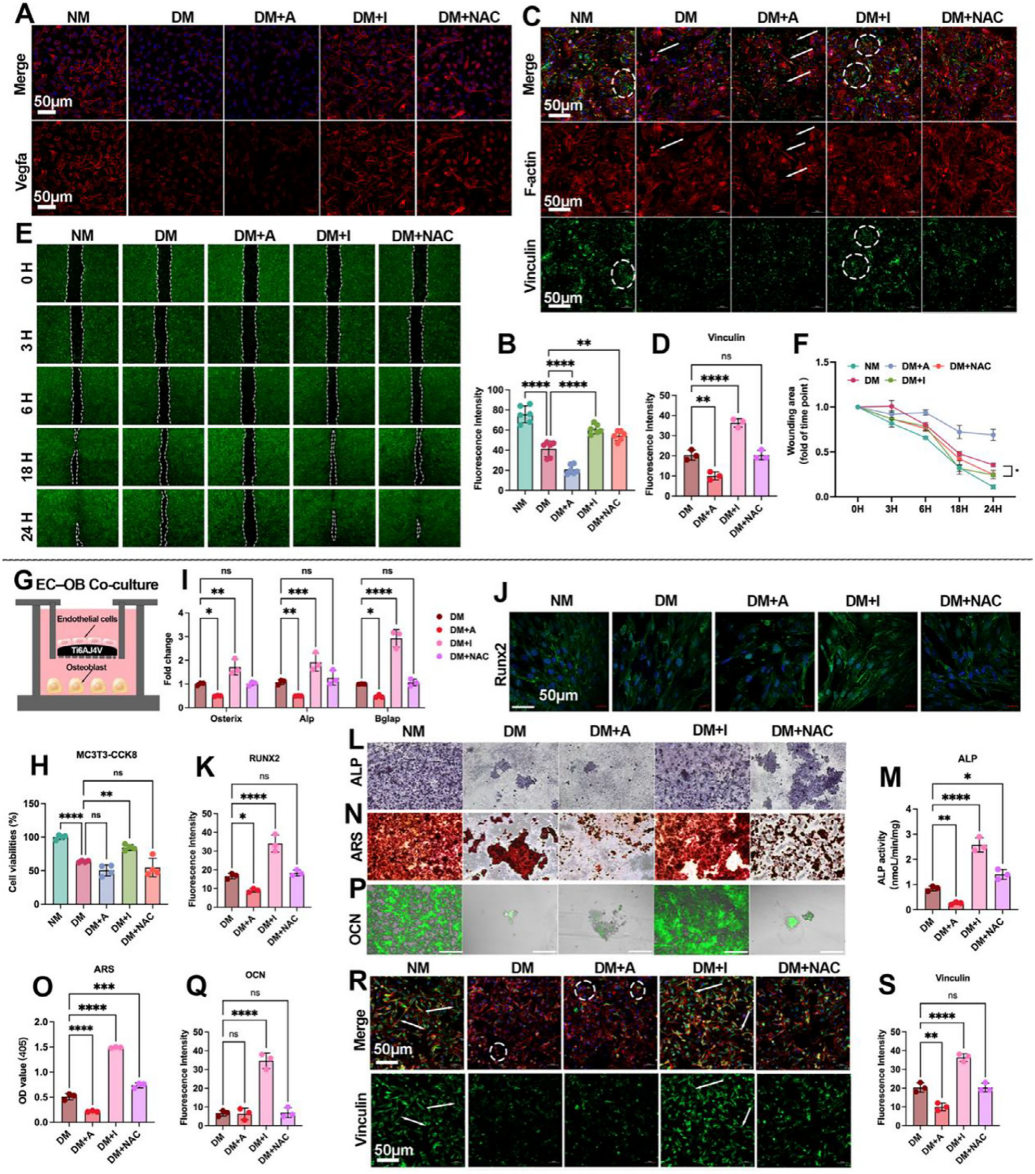
Pharmacologic NOX2 modulation alters endothelial functions and rescues osteogenesis in EC–OB co‐culture on Ti. A,B) immunofluorescenceand and quantification of VEGFA. C) immunofluorescence of ECs adhesion and morphology on day 3: F‐actin (phalloidin; red), vinculin (green), nuclei (DAPI; blue). D) quantification of vinculin. E) Representative wound‐healing images; Dashed lines mark wound borders at 0 h. F) Quantification of relative wound area at 24 h. G) Schematic of the Transwell co‐culture (bEnd.3 ECs with MC3T3‐E1 osteoblasts) on Ti; pharmacologic treatments: TMF (NOX2 agonist) and GSK2795039 (NOX2 inhibitor). H) CCK8 proliferation of osteoblasts. I) qPCR for osteogenic maker (Osterix, Alp, Bglap). J,K) Runx2 immunofluorescence and quantification. L,M) ALP staining and quantification (day 14). N,O) ARS staining and quantification (day 21). P,Q) OCN immunofluorescence and quantification (day 28). R,S) Osteoblast adhesion/morphology on Ti and quantification of adherent cells; arrowheads mark distorted F‐actin; dashed circles denote intracellular vinculin trafficking. Scale bar: 50 µm (A–C, J, R), 200 µm (L, N, P). Data presentation: mean ± SD; *n* = as indicated in panels. Statistics: one‐way ANOVA with Tukey's post hoc test for ≥ 3 groups (and two‐tailed Student's t‐tests for pairwise comparisons when applicable), as appropriate depending on data normality; *P* < 0.05 (*), *P* < 0.01 (**), *P* < 0.001 (***), *P* < 0.0001 (****); ns, not significant.

Under diabetic conditions, endothelial cells (bEnd.3) on Ti displayed a broad functional deficit that tracked with NOX2 activity. Endothelial viability was reduced in DM and further suppressed by the NOX2 agonist TMF (DM+A), whereas the NOX2 inhibitor GSK2795039 (DM+I) restored viability toward NM; by contrast, NAC did not improve and even lowered viability (Figure S4, Supporting Information). Pro‐angiogenic signaling was reduced in DM, as VEGFA immunofluorescence decreased relative to NM and was further suppressed by the NOX2 agonist TMF, whereas GSK2795039 a (and partially NAC) increased VEGFA (**Figure** **3**A,B). At the transcript level, Vegfa mRNA paralleled the immunofluorescence results: it was reduced in DM relative to NM, further suppressed by TMF, increased above NM by GSK2795039, and only partially rescued by NAC (Figure S5, Supporting Information). Cytoskeletal/adhesion organization was likewise impaired: DM and DM+A cells showed distorted F‐actin bundles with intracellular vinculin puncta, while DM+I (and NAC) restored well‐spread morphology and peripheral vinculin (Figure 3C), with a significant increase in vinculin intensity on quantification (Figure 3D). In scratch assays on Ti, migration was slowed in DM, exacerbated by TMF, and rescued by GSK2795039 (and NAC), as reflected by smaller relative wound area at 24 h (Figure 3E,F). Together, these data show that increasing NOX2 activity worsens—whereas inhibiting NOX2 improves—EC viability, pro‐angiogenic output, adhesion architecture, and motility on Ti.

Before co‐culture, Annexin V/PI flow cytometry of MC3T3 monocultures on Ti showed higher late apoptosis in DM versus NM and a further increase with TMF; notably, GSK2795039 did not reduce apoptosis and NAC showed no significant decrease (Figure S6, Supporting Information). These baseline data reinforce an endothelial‐centric mechanism and motivate EC–OB co‐culture assays on Ti.

To test whether endothelial NOX2 status constrains osteogenesis, we used a Transwell co‐culture of bEnd.3 (upper insert) and MC3T3‐E1 osteoblasts (lower compartment) on Ti with the same pharmacologic manipulations (Figure 3G). OB proliferation (CCK‐8) was reduced by DM and further decreased by TMF, while GSK2795039 (and NAC) restored proliferative capacity toward NM (Figure 3H). Early osteogenic transcription was suppressed in DM and enhanced by NOX2 inhibition, as Osterix, Alp, and Bglap mRNA increased in DM+I versus DM (Figure 3I). Consistently, RUNX2 immunofluorescence at day 3 rose in DM+I relative to DM (Figure 3J,K). Functionally, ALP staining/activity at day 14 and matrix mineralization (ARS) at day 21 were both lowest in DM/DM+A and rescued by GSK2795039 (and NAC) (Figure 3L–O). Late osteogenic protein OCN followed the same pattern (Figure 3P,Q). Finally, OB adhesion/morphology on Ti mirrored the EC phenotype: DM and TMF produced disorganized actin with reduced peripheral vinculin, whereas NOX2 inhibition restored a well‐spread, vinculin‐rich adhesome and increased adherent cell numbers (Figure 3R,S). Collectively, across EC and EC–OB co‐culture contexts on Ti, pharmacologic NOX2 activation (TMF) consistently aggravates oxidative‐stress–linked dysfunction, while NOX2 inhibition (GSK2795039) reverses deficits in EC angiogenic signalling, adhesion and migration, and rescues osteoblast proliferation, osteogenic gene expression, matrix maturation/mineralization, and late markers. These findings position NOX2 as a tractable upstream target to restore a pro‐angiogenic, pro‐osteogenic milieu under diabetic conditions.

### 2.4 Endothelial NOX2 Knockout Reverses EndMT and Restores Angiogenic Capacity

**Figure 4:**
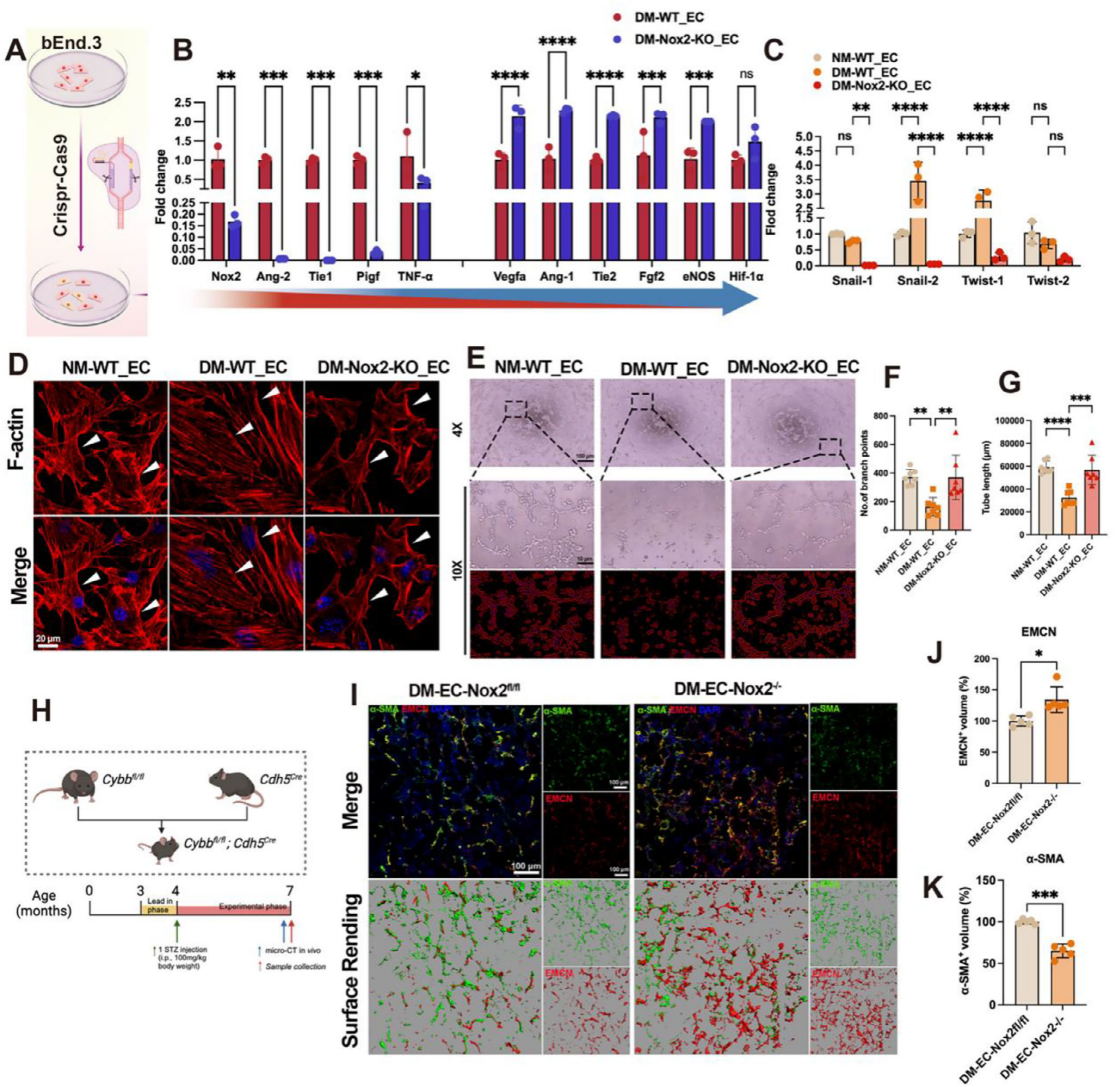
Endothelial NOX2 knockout (KO) suppresses EndMT and rescues angiogenic function. A) CRISPR/Cas9 KO validation in bEnd.3. B) qPCR of endothelial/angiogenic and inflammatory markers showing rescue by NOX2 KO under DM. C) qPCR of EndMT transcription factors (Snail1/2, Twist1/2). D) Morphology/cytoskeleton: phalloidin (F‐actin, red), DAPI (blue); arrowheads mark spindle‐like cells in DM‐WT_EC. E–G) Matrigel tube formation with quantification of branch points (F) and total tube length (G). H) Strategy for endothelial‐specific Nox2 deletion in vivo. I–K) BII immunofluorescence for α‐SMA (mesenchymal) and EMCN (endothelial) with quantification. Scale bars: 20 µm (D), 100 µm (E, I). Data presentation: mean ± SD; *n* = as indicated in panels. Statistics: two‐tailed Student's *t*‐tests for two‐group comparisons and one‐way ANOVA with Tukey's post hoc test for ≥3 groups, as appropriate depending on normality; *P* < 0.05 (*), *P* < 0.01 (**), *P* < 0.001 (***), *P* < 0.0001 (****); ns, not significant.

To test whether NOX2 is required for diabetes‐associated endothelial dysfunction, we generated a CRISPR/Cas9 NOX2 knockout (KO) in bEnd.3 cells (**Figure** **4**A). The gRNA targeted the mouse Nox2 locus (Figure S7A, Supporting Information), and immunoblotting confirmed ≈99% loss of NOX2 protein (Figure S7B, Supporting Information), establishing a high‐efficiency KO model.

In diabetic medium, wild‐type ECs (DM‐WT_EC) showed a shift away from an angiogenic phenotype, whereas NOX2 deletion (DM‐Nox2‐KO_EC) restored pro‐angiogenic/EC identity programs and dampened inflammatory cues (Figure 4B). Specifically, KO increased Vegfa, Ang‐1, Tie2, Fgf2, eNOS, and reduced Ang‐2 and TNF‐α relative to DM‐WT_EC (Hif‐1α trended similarly). In parallel, EndMT transcription factors were suppressed by KO: Snail1/2 and Twist1 were elevated by DM but significantly decreased in DM‐Nox2‐KO_EC, whereas Twist2 was unchanged (Figure 4C). Morphology and cytoskeleton. Consistent with EndMT, DM‐WT_EC adopted a spindle‐like, fibroblastoid morphology with pronounced stress fibers (phalloidin), while NOX2‐deficient cells retained a cobblestone endothelial appearance comparable to NM‐WT_EC (arrowheads, Figure 4D). Angiogenic function. On Matrigel, tubulogenesis was impaired in DM‐WT_EC versus NM‐WT_EC (Figure 4E). NOX2 KO rescued tube formation, increasing branch points (DM‐Nox2‐KO_EC: 369.60 ± 155.40 vs DM‐WT_EC: 164.00 ± 64.07) and total tube length (DM‐Nox2‐KO_EC: 56825.00 ± 12833.00 vs DM‐WT_EC: 32306.00 ± 6576.00) (Figure 4F,G); KO values were not significantly different from NM‐WT_EC, indicating near‐complete functional recovery.

To determine endothelial NOX2 necessity in the diabetic microenvironment, we generated EC‐specific Nox2 knockout mice (DM–EC‐Nox2^−/−^) using Cdh5‐Cre crossed to Nox2^fl/fl^ and compared with diabetic littermate controls (DM–EC‐Nox2^fl/fl^) (Figure 4H). At the BII, confocal immunofluorescence with 3D rendering revealed that DM–EC‐Nox2^−/−^ mice exhibited a denser EMCN⁺ endothelial network and reduced α‐SMA⁺ perivascular signal relative to controls (Figure 4I). Quantitatively, EMCN⁺ volume was increased and α‐SMA⁺ volume decreased in DM–EC‐Nox2^−/−^ (Figure 4J,K), recapitulating in‐vitro EndMT suppression and angiogenic rescue. Across cellular and in‐vivo settings, endothelial NOX2 is necessary for diabetes‐induced EndMT and loss of angiogenic capacity. Deleting NOX2 preserves endothelial identity, restores angiogenic programs and tubulogenesis, and alleviates mesenchymal transition at the BII.

### 2.5 Pharmacologic NOX2 Inhibition Restores Early Angiogenesis–Osteogenesis Coupling and Improves Osseointegration In Vivo

**Figure 5:**
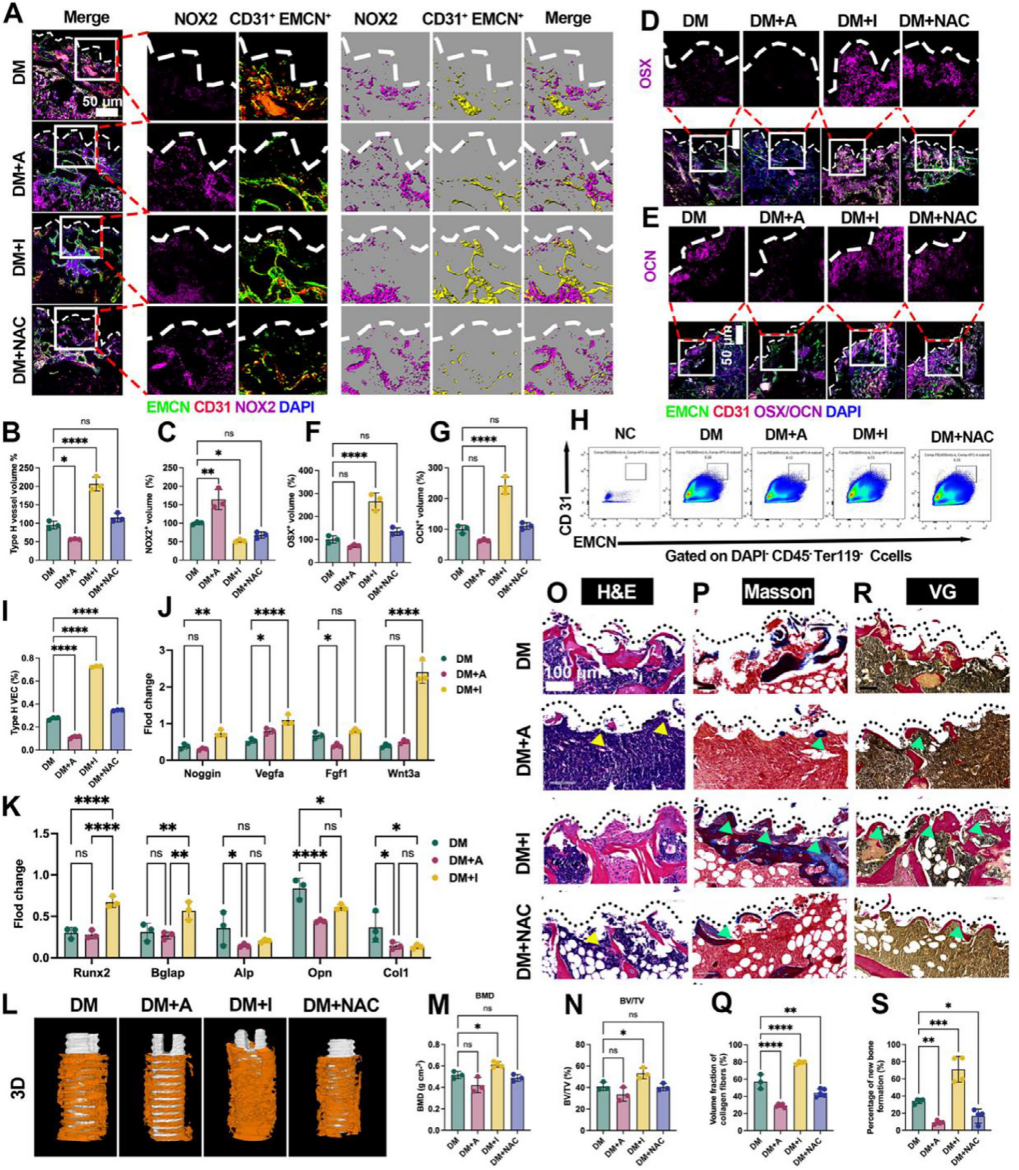
In vivo pharmacologic inhibition of NOX2 restores early coupling (2 weeks) and enhances osseointegration (8 weeks). A,D,E) Two‐week immunofluorescence around Ti implants showing NOX2 (A), OSX (D), OCN (E), CD31^+^EMCN^+^ (type‐H vessel) and DAPI, with 3D surface rendering; B,C,F,G) corresponding quantifications (type‐H, NOX2, OSX⁺, OCN⁺). H,I) Flow cytometry of CD31⁺EMCN⁺ endothelial cells: representative plots and group summary. J) qPCR of coupling cytokines (Noggin, Vegfa, Fgf1, and Wnt3a) and K) osteoblast genes (Runx2, Bglap, Alp, Opn, and Col1a1) in peri‐implant tissue. L) micro‐CT 3D reconstructions (implant, white; peri‐implant bone, yellow) with ROI; M,N) peri‐implant BMD and BV/TV. O) H&E of peri‐implant bone/fibrous tissue; P) Masson's trichrome with Q) collagen/mature bone fraction; R) Van Gieson with S) percentage of new bone around implants. Dashed line, implant–tissue interface. Scale bars: 50 µm (A, D, E), 100 µm (O, P, R). Groups: DM (vehicle), DM+A (TMF), DM+I (GSK2795039), DM+NAC. Data presentation: mean ± SD; *n* = as indicated in panels. Statistics: one‐way ANOVA with Tukey's post hoc test for ≥ 3 groups (and two‐tailed Student's *t*‐tests for pairwise comparisons when applicable), as appropriate depending on data normality; *P* < 0.05 (*), *P* < 0.01 (**), *P* < 0.001 (***), *P* < 0.0001 (****); ns, not significant.

To determine whether NOX2 is a tractable driver of the diabetic interface phenotype, Ti‐implanted diabetic mice received vehicle (DM), the NOX2 agonist TMF (DM+A), the NOX2 inhibitor GSK2795039 (DM+I), or the ROS scavenger NAC (DM+NAC). Immunofluorescence around the BII confirmed effective target engagement: NOX2 signal decreased with DM+I (52.50 ± 3.69; *P* < 0.05 vs DM) and increased with DM+A (164.20 ± 27.47; *P* < 0.01) (**Figure** **5**A–C). Importantly, type‐H endothelium (CD31⁺EMCN⁺) rose markedly with DM+I (206.30 ± 18.68; *P* < 0.0001) but fell with DM+A (56.70 ± 1.09 vs 95.08 ± 10.65 in DM; *P* < 0.05) (Figure 5A,B). Osteolineage populations at the interface followed the same pattern: OSX⁺ osteoprogenitors and OCN⁺ mature osteoblasts were both higher in DM+I than DM (OSX⁺ 265.3 ± 37.27; OCN⁺ 242.2 ± 27.46), whereas TMF worsened the deficits (Figure 5D–G).

Flow cytometry using CD31 and EMCN gates corroborated these trends: the fraction of type‐H ECs was lowest with DM+A (0.11 ± 0.01; *P* < 0.0001 vs DM) and increased with DM+I (0.73 ± 0.01; *P* < 0.0001) and DM+NAC (0.35 ± 0.01 versus 0.27 ± 0.01 in DM; *P* < 0.0001) (Figure 5H,I). At the molecular level, peri‐implant qPCR showed that DM+I up‐regulated coupling mediators—Noggin (0.73 ± 0.09 vs 0.38 ± 0.07; P<0.01), Vegfa (1.10 ± 0.14 vs 0.53 ± 0.07; *P* < 0.0001), Fgf1 (2.41 ± 0.31 vs 0.40 ± 0.05; *P* < 0.0001) (Figure 5J)—and boosted osteoblast genes Runx2 (0.67 ± 0.07 vs 0.30 ± 0.07; *P* < 0.0001) and Bglap (0.57 ± 0.11 vs 0.31 ± 0.11; *P* < 0.01) relative to DM (Figure 5K). Together, these data show that pharmacologic NOX2 inhibition rapidly restores endothelial integrity and pro‐osteogenic signalling, consistent with re‐coupling of angiogenesis and osteogenesis at the interface.

We next assessed whether early rescue translates into improved bone regeneration and functional osseointegration. micro‐CT 3D reconstructions revealed greater peri‐implant bone in DM+I versus DM (Figure 5L). Quantitatively, the peri‐implant BMD and BV/TV within the ROI were increased in DM+I compared with DM (Figure 5M,N), indicating partial restoration of osseointegration. Histological analyses corroborated these findings: H&E showed substantial bone encasing implants after DM+I, whereas DM and especially DM+A displayed fibrous replacement (Figure 5O). Masson's trichrome confirmed enhanced matrix maturation with DM+I (higher fraction of mature collagen/mineralized tissue) and a further decline with TMF (Figure 5P,Q). Van Gieson likewise demonstrated increased collagen‐rich new bone after DM+I, with histomorphometry showing a significantly greater percentage of new bone around implants relative to DM (Figure 5R,S). Collectively, NOX2 inhibition not only re‐couples the early vascular‐osteogenic axis but also improves osseointegration at the endpoint, highlighting NOX2 as a therapeutically actionable node for restoring BII integrity under diabetic conditions.

### 2.6 Endothelial‐Specific NOX2 Deletion Enhances Angiogenesis–Osteogenesis Coupling and Mitigates Diabetic Bone Disease In Vivo

**Figure 6:**
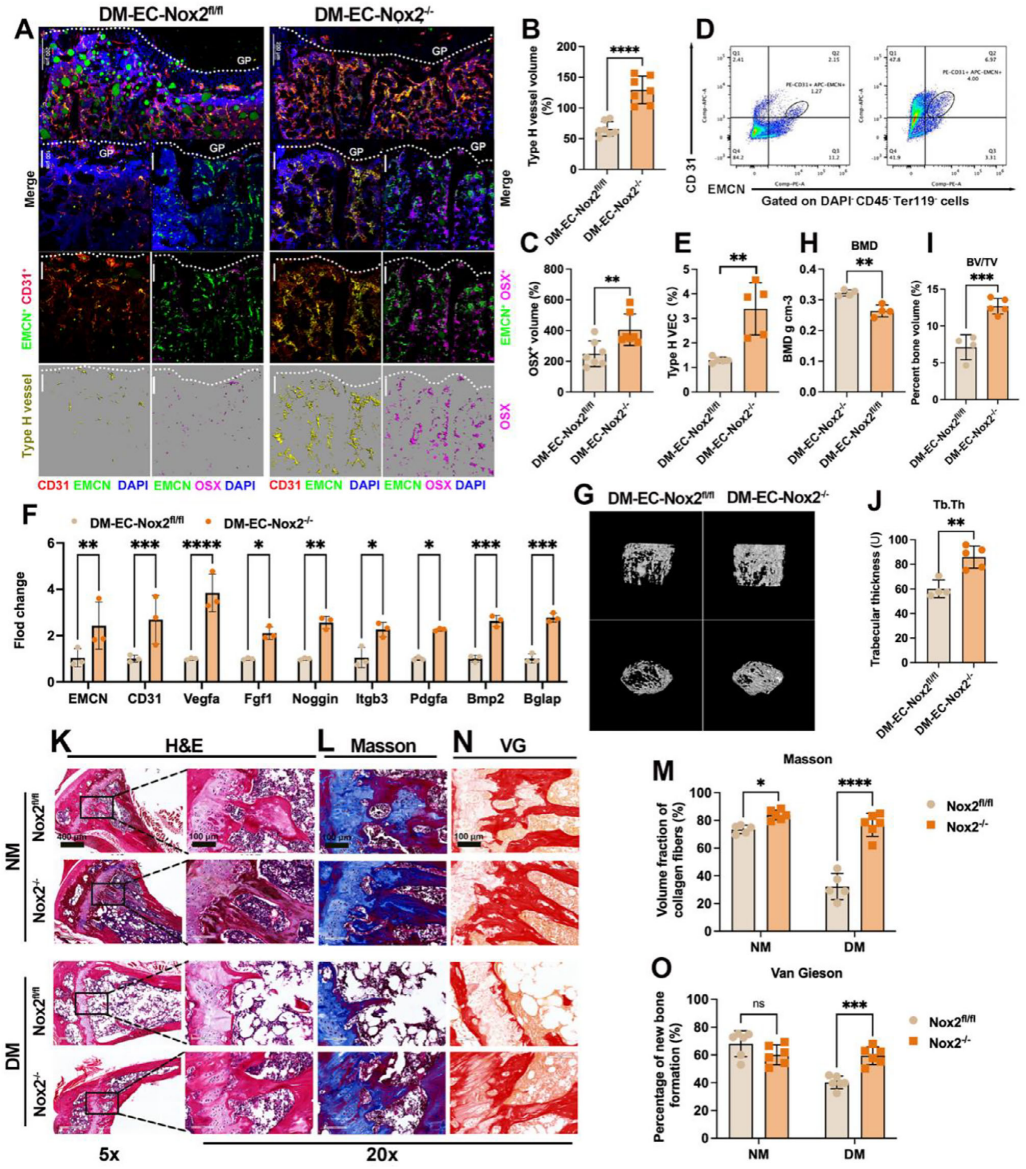
Endothelial‐specific Nox2 deletion (EC‐Nox2^−/−^) improves coupling and skeletal outcomes in diabetes. A) Confocal images and 3D renderings of tibial metaphysis in DM–EC‐Nox2^fl/fl^ and DM–EC‐Nox2^−/−^ mice showing CD31 (red), EMCN (green), OSX (magenta), DAPI (blue); dotted lines, growth plate (GP). B,C) Histomorphometry of type‐H vessel volume and OSX⁺ volume. D,E) Flow cytometry of CD31⁺EMCN⁺ ECs (type‐H): representative plots and group summary. F) qPCR (DAPI^−^CD45^−^Ter119^−^ cells): Nox2 downregulation with concomitant increases in EMCN, CD31, Vegfa, Fgf1, Noggin, Itgb3, Pdgfa, Bmp2, Bglap. G) micro‐CT reconstructions (3D and 2D) with ROI. H–J) BMD, BV/TV, and Tb.Th. K) H&E; L) Masson's trichrome; M) collagen fiber volume fraction (Masson) in NM and DM. (N) Van Gieson staining near GP; (O) percentage of newly formed bone. Scale bars: 100 µm (A), 5× = 400 µm and 20× = 100 µm (K, L, N). Data presentation: mean ± SD; *n* = as indicated in panels. Statistics: two‐tailed Student's *t*‐tests for two‐group comparisons and one‐way ANOVA with Tukey's post hoc test for ≥3 groups, as appropriate depending on normality; *P* < 0.05 (*), *P* < 0.01 (**), *P* < 0.001 (***), *P* < 0.0001 (****); ns, not significant.

To determine whether endothelial NOX2 is required for the diabetic coupling defect, we analyzed tibial metaphyses (no implant) from diabetic mice with endothelial Nox2 deletion (DM–EC‐Nox2^−/^^−^) and diabetic littermate controls (DM–EC‐Nox2^fl/fl^). Confocal imaging with 3D surface rendering showed a denser CD31⁺EMCN⁺ (type‐H) network and more adjacent OSX⁺ osteoprogenitors in DM–EC‐Nox2^−/^^−^ than controls (**Figure** **6**A). Histomorphometry confirmed higher type‐H vessel volume (129.3 ± 22.36 vs 65.98 ± 11.85) and increased OSX⁺ volume (405.3 ± 102.4 vs 249.2 ± 83.35) in DM–EC‐Nox2^−/^^−^ (Figure 6B–C). Flow cytometry corroborated these findings, with a greater fraction of type‐H ECs (3.39 ± 1.06% vs 1.30 ± 0.12%) in DM–EC‐Nox2^−/^^−^ (Figure 6D,E). qPCR of DAPI^−^CD45^−^Ter119^−^ cells validated Nox2 knockdown (0.16 ± 0.02 vs 1.01 ± 0.13) and up‐regulation of pro‐coupling and osteogenic genes in DM–EC‐Nox2^−/^^−^—EMCN, CD31, Vegfa, Fgf1, Noggin, Itgb3, Pdgfa, Bmp2, Bglap (Figure 6F). These data indicate that endothelial NOX2 ablation restores the type‐H vascular niche and re‐couples angiogenesis and osteogenesis under diabetic conditions.

We next asked whether this vascular rescue translates to bone quality. micro‐CT revealed a denser trabecular network in DM–EC‐Nox2^−/^^−^ compared with diabetic controls (Figure 6G), with increased BMD (0.32 ± 0.01 vs 0.26 ± 0.02), BV/TV (12.69 ± 1.01 vs 7.12 ± 1.70), and Tb.Th (85.91 ± 9.03 vs 60.10 ± 7.17) (Figure 6H–J). Histological analyses supported these improvements: H&E showed preservation of trabeculae in DM–EC‐Nox2^−/^^−^ (Figure 6K); Masson's trichrome demonstrated greater collagen/mineralized matrix in DM–EC‐Nox2^−/^^−^ both in NM and DM cohorts (NM: 83.92 ± 3.47% vs 73.47 ± 3.05%; DM: 76.76 ± 8.29% vs 32.27 ± 9.47%) (Figure 6L,M); and Van Gieson staining revealed increased newly formed bone near the growth plate in EC‐Nox2^−/−^, with histomorphometry confirming higher neogenesis in DM (EC‐Nox2^−/^^−^ > EC‐Nox2^fl/fl^; NM groups, n.s.) (Figure 6N,O). Together, endothelial‐specific Nox2 deletion rebuilds the type‐H/OSX⁺ niche and improves trabecular metrics in diabetic bone, positioning NOX2 as a regulator—and therapeutic target—of diabetic bone disease.

### 2.7 Transcriptomic Profiling Corroborates EndMT Suppression and Pathway Reversals upon NOX2 Loss

**Figure 7:**
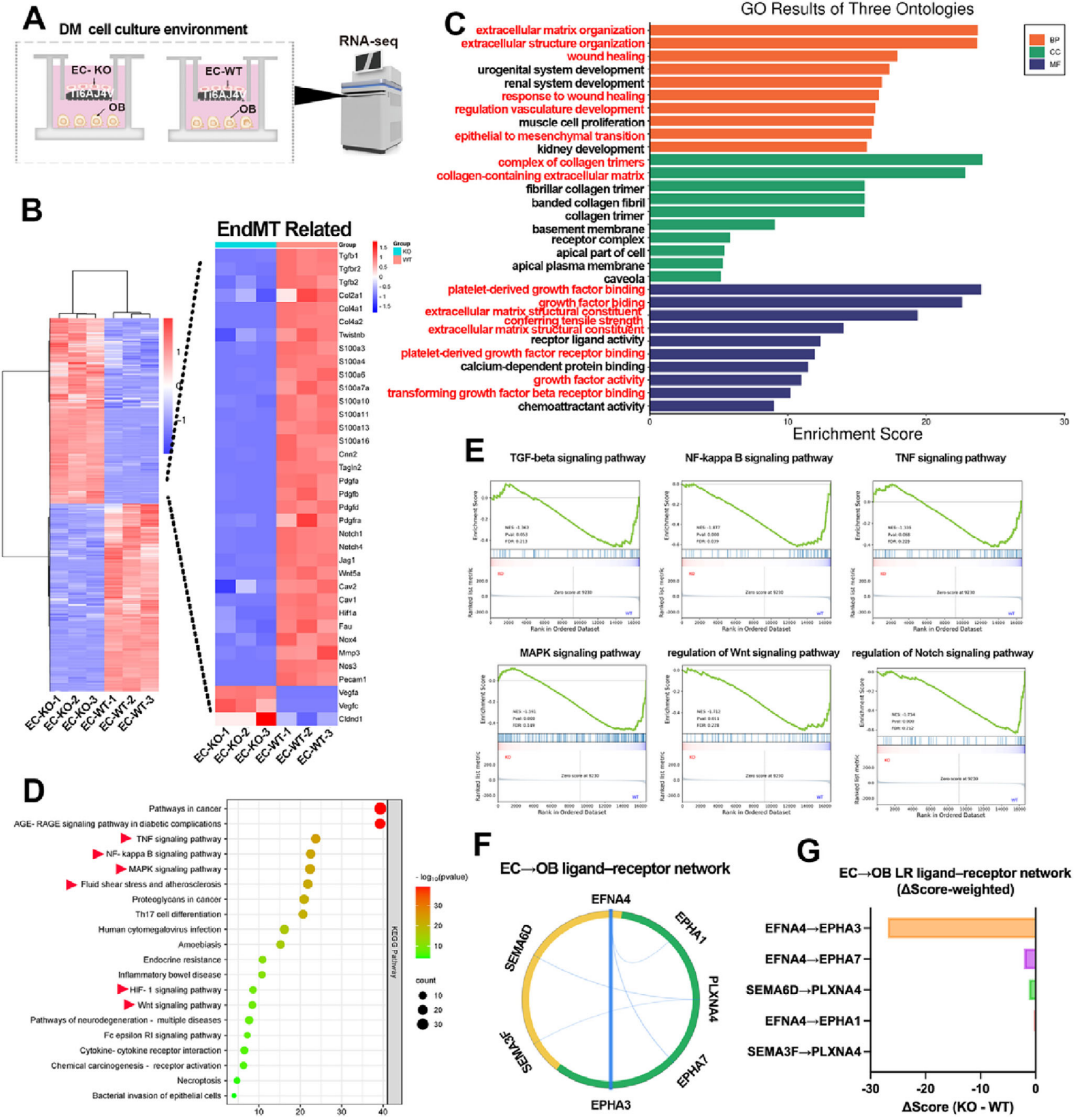
Transcriptomic profiling corroborates EndMT suppression and pathway reversals upon NOX2 loss. A) Experimental design: EC–OB Transwell co‐culture on Ti under diabetic conditions followed by RNA‐seq. Endothelial cells were wild type (EC‐WT) or NOX2‐knockout (EC‐KO); osteoblasts were WT but exposed to EC‐WT or EC‐KO and are denoted OB‐WT and OB‐KO, respectively. B) Heat map of differentially expressed genes (scaled per gene), with an inset highlighting EndMT‐related genes. C) Gene Ontology enrichment across BP/CC/MF for EC‐KO versus EC‐WT, showing enrichment in extracellular matrix/structure organization, receptor/growth‐factor binding, and membrane components. D) KEGG pathway enrichment bubble plot for EC‐KO versus EC‐WT. (OB‐axis GO/KEGG enrichments are provided in Supplementary Figures.) E) Representative GSEA plots showing negative enrichment (NES < 0) of EndMT‐linked and inflammatory pathways (TGF‐β, NF‐κB, TNF, and MAPK) and guidance programs (Wnt, Notch) in EC‐KO relative to EC‐WT; NES and FDR are indicated. F) EC to OB ligand–receptor chord network derived from replicate means (EC ligands, yellow; OB receptors, green). G) Bar plot of the top LR edges.

To integrate the pharmacologic and genetic evidence mechanistically, we performed bulk RNA‐seq on the EC–OB Transwell co‐culture on Ti under diabetic conditions. Endothelial cells were either wild type (EC‐WT) or NOX2‐KO (EC‐KO); osteoblasts in both arms were WT but exposed to conditioned signaling from EC‐WT or EC‐KO (denoted OB‐WT and OB‐KO, respectively; **Figure** **7**A). With |log_2_FC| > 1 and Benjamini–Hochberg FDR q < 0.001, EC‐KO versus EC‐WT yielded thousands of DEGs whereas OB‐KO versus OB‐WT yielded hundreds, consistent with a secondary, paracrine remodeling on the OB side (Figure S8, Supporting Information). Replicates clustered tightly and separated by compartment and genotype (Figure S9, Supporting Information), supporting robust contrasts.EC‐KO versus EC‐WT yielded thousands of DEGs, whereas OB‐KO versus OB‐WT yielded hundreds, consistent with a secondary, paracrine remodeling on the OB side. Replicates clustered tightly (heatmap; Figure 7B), supporting robust contrasts.

#### 2.7.1 EC Axis—EndMT Programs Collapse, Endothelial Identity Restored

Heatmap inspection highlighted a coordinated down‐shift of EndMT drivers and mesenchymal/ECM modules in EC‐KO (e.g., TGF‐β axis, collagens, cytoskeletal/contractile markers), alongside higher endothelial‐identity/angiogenic transcripts (e.g., Pecam1, Nos3, Vegfa/Vegfc), aligning with imaging‐level EndMT suppression. GO analysis of EC‐KO‐up genes emphasized extracellular matrix/structure organization, collagen‐containing ECM, growth‐factor binding and receptor binding, and membrane‐associated components (Figure 7C). KEGG and GSEA analyses revealed coherent negative enrichment (NES < 0; FDR < 0.05) of TGF‐β, NF‐κB, TNF, MAPK, and the Wnt/Notch programs in EC‐KO relative to EC‐WT (Figure 7D,E). The canonical JAK–STAT signaling pathway itself did not reach statistical significance (FDR ≥0.05); however, several representative components of this axis (Jak1, Jak3, Socs3, and Il6ra) showed directionally concordant down‐regulation trends in EC‐KO (Table S1 and Figure S15, Supporting Information). The heatmap visualization further demonstrates consistent attenuation of multiple JAK–STAT elements (JAK–STAT1/2/3/5 subunits and regulatory mediators) in EC‐KO samples, aligning with suppression of cytokine‐driven inflammatory signaling. This gene‐level pattern supports an overall attenuation of endothelial activation upon NOX2 loss and is consistent with the down‐regulation of other inflammatory cascades such as TGF‐β, NF‐κB, and MAPK pathways described above. Our transcriptomic analysis revealed that NOX2 deletion in ECs significantly down‐regulated key inflammatory pathways including TGF‐β, NF‐κB, MAPK, Wnt, and Notch, contributing to the suppression of EndMT‐like endothelial reprogramming and restoration of endothelial identity. These findings are consistent with the observed suppression of EndMT markers (Snail1, Twist1/2) and the restoration of angiogenesis‐associated genes (e.g., Vegfa, Ang‐1) at the transcriptomic and protein levels.

#### 2.7.2 OB Axis—Paracrine ECM/Adhesion Rewiring with Stress–Translation Compensation

Consistent with an EC‐driven cueing effect, OB DEGs showed selective remodeling of matrix/adhesion and guidance pathways. A focused OB heatmap highlighted broad reductions in ECM/adhesion transcripts in OB‐KO—collagens, laminins, integrins (Itga1/7/11), fibronectin (Fn1), and Adamts proteases—compatible with attenuation of guidance/adhesion signaling (Figure S10, Supporting Information). Enrichment analyses supported this view. Among down‐regulated OB genes we observed KEGG terms such as ECM–receptor interaction, focal adhesion, regulation of actin cytoskeleton, PI3K–AKT, MAPK, axon guidance (Figure S13, Supporting Information), and GO terms involving membrane/cytoskeleton and lipid/sterol metabolic processes (Figure S11, Supporting Information). Conversely, up‐regulated OB genes were enriched for cytoplasm/nucleus/ER/ribosomal constituents and processes related to translation, ER protein processing/UPR, chromatin organization, cell‐cycle control, and apoptosis regulation (Figures S12 and S14, Supporting Information). Together, these OB‐side enrichments suggest a secondary ECM/adhesion de‐emphasis with compensatory protein‐handling/translation and stress‐response programs when osteoblasts are exposed to NOX2‐deficient endothelium.

#### 2.7.3 Ligand–Receptor (LR) Rewiring from EC to OB

Using replicate‐mean expression to score LR pairs, the EC to OB network revealed reduced guidance/adhesion edges after NOX2 loss—most notably EFNA4–EPHA1/3/7 and SEMA3F/SEMA6D–PLXNA4—when ranked by |ΔScore| (ΔScore = KO − WT) (Figure 7F,G). This LR attenuation dovetails with the OB KEGG/GO signatures above, indicating that NOX2 deletion in ECs rewires ephrin/plexin guidance and adhesion cues to osteoblasts, congruent with restored coupling biology.

## 3 Discussion

**Figure 8:**
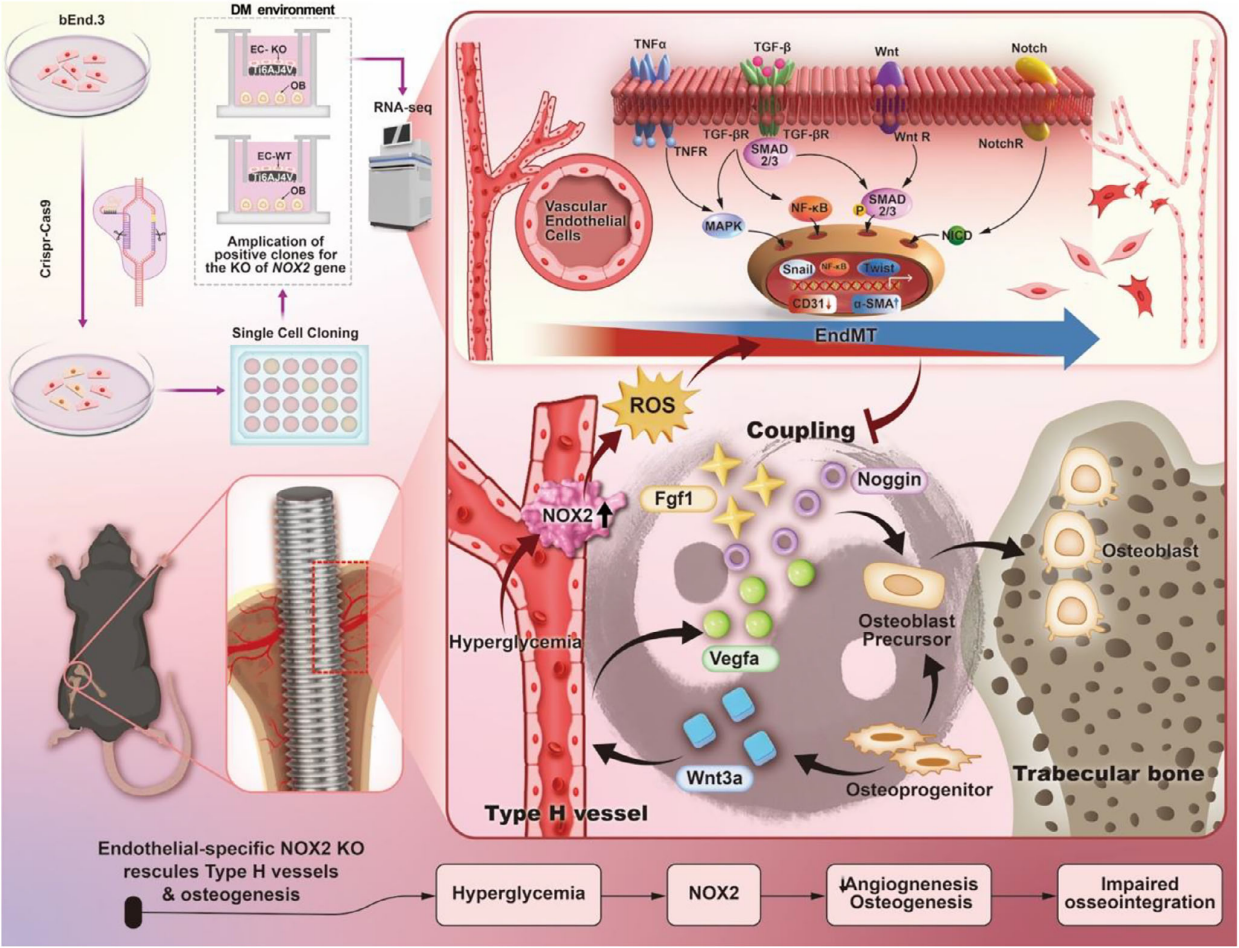
Schematic illustration of NOX2‐mediated EndMT impairs angiogenesis‐osteogenesis coupling and osseointegration under diabetic conditions. Endothelial NOX2–driven oxidative stress triggers EndMT and loss of type‐H vessels, uncoupling angiogenesis from osteogenesis at the diabetic implant interface. Blocking NOX2 restores endothelial identity, re‐couples vessel–bone signaling, and improves osseointegration. Transcriptomics confirm pathway reversals and reduced EC to OB ephrin/plexin guidance, highlighting NOX2 as an actionable interface target.

In this study, we identify NOX2‐driven oxidative stress as a central driver of impaired osseointegration in diabetic bone disease, acting through EndMT‐mediated uncoupling of angiogenesis and osteogenesis at the BII. A summary model is shown in **Figure** **8**. Using both pharmacologic inhibition and endothelial‐specific Nox2 deletion, we show that elevated NOX2 in the diabetic microenvironment promotes ROS accumulation, amplifies EndMT programs, and depletes type‐H endothelium—a vascular subtype that supports osteoprogenitor recruitment and bone formation. Functionally, NOX2 blockade or deletion restored vascular homeostasis, restrained EndMT progression, and rescued angiogenic and osteogenic capacity in vitro and in vivo. These improvements translated into enhanced peri‐implant bone volume, trabecular microarchitecture, and bone–implant contact. Transcriptomic profiling further revealed pathway reversals with NOX2 loss—downregulation of EndMT‐linked signaling (TGF‐β, NF‐κB/TNF, MAPK, Wnt/Notch) alongside upregulation of programs related to extracellular‐matrix organization, collagen assembly, and vascular signaling. Collectively, our data position NOX2 as a tractable upstream target to re‐establish a functional osteogenic niche under diabetic conditions.

Mechanistically, our findings position NOX2 as the molecular link between hyperglycemia‐driven oxidative stress and endothelial instability that uncouples angiogenesis from osteogenesis. Hyperglycemia and inflammatory signalling activate NOX isoform—particularly NOX2 in vascular endothelial cells—leading to excess ROS production.^[^ ^33^, ^34^, ^35^ ^]^ Beyond oxidative damage, ROS act as second messengers that promote EndMT through canonical fibrotic/inflammatory cascades, including TGF‐β/SMAD, Wnt/β‐catenin, NF‐κB, and MAPK.^[^ ^36^, ^37^, ^38^ ^]^ Consistent with this framework, our transcriptome data show that NOX2 deletion downregulated EndMT pathway genes while restoring endothelial‐identity and vessel‐stability programs. As type H endothelium (CD31^+^EMCN^+^) orchestrates osteoprogenitor recruitment and new bone formation,^[^ ^8^, ^39^ ^]^ NOX2‐driven EndMT and the attendant ECM remodeling plausibly compromise type H identity and abundance under diabetes, depriving the interface of required vascular support for remodeling/mineralization. Notably, we also observed of ECM signatures at the BII upon NOX2 inhibition/deletion, a process relevant to preventing fibrotic encapsulation and interface instability.^[^ ^40^ ^]^ Collectively, these data delineate a ROS–EndMT–type H axis governed by NOX2 that regulates the angiogenic–osteogenic interface during implant integration. While NOX2 dysregulation has been implicated in diabetic microangiopathy of cardiovascular and renal beds,^[^ ^41^, ^42^, ^43^ ^]^ its role in skeletal microvasculature and implant—interface remodeling has remained underexplored; our study covers this gap and provides a mechanistic basis for anti‐ oxidative vascular targeting to restore osseointegration in diabetic.

Prior work has documented detrimental effects of diabetes on bone metabolism and implant outcomes; however, the endothelial contribution to poor osseointegration has been less precisely defined at the BII. It was well known that systemic factors—advanced glycation end‐products, chronic inflammation, and impaired osteoblast differentiation—contribute to bone fragility,^[^ ^44^, ^45^, ^46^, ^47^ ^]^ yet they do not fully explain the local failure of osteoangiogenic coupling. Recent advances highlight angiogenesis–osteogenesis coupling via type H endothelium as a prerequisite for bone homeostasis and implant integration,^[^ ^13^, ^14^ ^]^ but the connection between vascular oxidative injury, endothelial phenotypic transition, type H vascular endothelium attrition, and interface failure has been unclear. Here we identify NOX2‐mediated EndMT as a central driver of vascular rarefaction and uncoupling loss in the diabetic implant niche. Compared with surface‐focused strategies (e.g., nanotopography, bioactive coatings, or ion doping) to improve osseointegration under diabetes,^[^ ^48^, ^49^ ^]^ our approach actively modulates the biological microenvironment by targeting a defined endothelial pathway. We show that genetic or pharmacological NOX2 blockade reduces ROS and restores both endothelial phenotype and osteogenic capacity, supporting a mechanistically grounded intervention that its compatible with systemic or local delivery. Clinically, NOX2 inhibition could complement biomaterial design—e.g., local inhibitors delivery, siRNA‐functionalized coatings, or NOX2‐suppressive elements integrated into hydrogels/porous scaffolds—to create interface‐modulating therapies tailored for diabetic patients.^[^ ^50^, ^51^ ^]^ This endothelial‐targeted axis may be generalizable across orthopedic, and dental implants.

Despite these strengths, several limitations warrant consideration. First, this study employed a type 2 diabetic mouse model (high‐fat diet combined with streptozotocin, HFD + STZ) with femoral implantation and 2‐ and 8‐week observation windows, which define the current experimental scope. The high‐fat‐diet/ streptozotocin mouse model recapitulates key aspects of T2DM but cannot capture the heterogeneity and chronicity of human disease; validation in larger animal (e.g., minipigs or non‐human primates) with load‐bearing implants is desirable for translation. Second, our endothelial‐specific NOX2 knockout isolates the vascular contribution, yet diabetes likely perturbs BII‐resident cells (osteoblasts, osteoclasts, pericytes, macrophages). Future single‐cell transcriptomics or spatial transcriptomics could resolve multicellular crosstalk and spatiotemporal dynamics of the diabetic niche. Third, although pharmacological inhibitors and gene deletion establish causality, the long‐term safety, dosing, and delivery efficiency of NOX2‐targeted therapies require evaluation. Localized systems—NOX2‐siRNA hydrogels or titanium coatings— should be optimized for durability, off‐target minimization, and mechanical compatibility. In addition, localized periosteal micro‐injection used in this study demonstrates the feasibility and safety advantage of confined drug exposure at the bone–implant interface. Such local delivery minimizes systemic spillover and provides a practical basis for developing implant‐surface functionalization strategies or hydrogel depots for controlled NOX2 inhibition in future translational applications. Finally, this work focuses on the vascular axis; integrative strategies that combine ROS scavenging, immunomodulation, and stem‐cell recruitment within smart biomaterials may further enhance outcomes in diabetic implantology. In sum, our findings identify NOX2 as a tractable molecular node linking endothelial dysfunction to interface failure and outline a path toward vascular‐centric, interface‐modulating therapies for clinical translation.

## 4 Conclusion

This study identifies a mechanism whereby NOX2‐driven oxidative stress promotes EndMT and uncouples angiogenesis from osteogenesis at the BII under diabetic conditions. Across pharmacologic inhibition and endothelial‐specific Nox2 deletion, targeting NOX2 restored endothelial identity and type H endothelium, attenuated EndMT, and rescued interface angiogenic and osteogenic functions—yielding early recoupling at 2 weeks and improved osseointegration at 8 weeks. Transcriptomic profiling corroborated pathway reversals consistent with EndMT attenuation.

These findings position NOX2 as an actionable mediator of vascular dysfunction at the BII and provide a mechanistic rationale for interface‐focused interventions that temper oxidative stress and preserve endothelial integrity. Translational avenues include local NOX2‐inhibitor delivery, siRNA‐functionalized or anti‐EndMT coatings, and microenvironment‐responsive hydrogels/porous scaffolds tailored to diabetic niches. As diabetes prevalence rises, aligning molecular targeting with biomaterial design offers a mechanism‐based path to restore angiogenesis–osteogenesis coupling and strengthen osseointegration in high‐risk patients. Together, these data support vascular‐centric, NOX2‐targeted strategies to improve implant outcomes in diabetes.

## 5 Experimental Section

### Preparation of the Ti6Al4V Specimens

The Ti6Al4V alloy was machined into circular disks (10 mm diameter, 1 mm thickness) and screws (1 mm diameter, 2.5 mm length) by Dabo Medical Equipment Co., Ltd., (Xiamen, China). Circular disks were used for in vitro experiments and screws for in vivo implantation studies. All Ti specimens were mechanically polished and cleaned ultrasonically in ethanol and deionized water, without any additional surface treatment or coating, to ensure uniform surface finish across samples.

### High‐Hat‐Diet/Streptozotocin‐Induced Diabetic Mice

Male C57BL/6 mice (12 weeks old) were housed under specific‐pathogen‐free conditions (24 ± 2 °C; 55% ± 5% humidity). A T2DM mouse model was established as previous described.^[^ ^52^ ^]^ After 1 week of acclimatization, mice were randomly assigned to non‐diabetic (NM) and diabetic (DM) groups. DM mice received a high‐fat‐diet (HFD, D12492) and a single intraperitoneal injection of streptozotocin (STZ, 100 mg kg^−1^), whereas NM mice received vehicle only. Following the 12 weeks HFD, mice with blood glucose levels >13.8 mmol L^−1^ were classified as diabetic.

### Implantation Surgery and Pharmacologic Treatments

Titanium screws implantation into the right femurs was performed under aseptic conditions as previously described.^[^ ^18^ ^]^ Briefly, through a ≈5 mm medial parapatellar approach, the distal femoral metaphysis was opened to access the medullary canal and a Ti6Al4V screw was advanced beneath the articular surface. The extensor mechanism was reconstructed and the wound closed in layers; perioperative analgesia, a 3‐day antibiotic course, and monitoring followed institutional guidelines.

### Mice were Randomized to Groups with Blinded Outcome Assessment

NM served as non‐diabetic controls. DM mice were assigned to 4 groups (*n* = 6 per group): DM (vehicle), DM + A (4′,6,7‐Trimethoxyisoflavone; NOX2 agonist, 4 mm), DM + I (GSK2795039; NOX2 inhibitor, 4 mm), and DM + NAC (Acetylcysteine; ROS inhibitor, 4 mm). Beginning on the day of implantation, 10 µL of the designated agent was injected periosteally at the distal femur every 3 days for 2 weeks; NM and DM groups received saline. Cohorts were euthanized at 2 weeks (early coupling) or 8 weeks (osseointegration); the 8‐week cohort received the same 2‐week dosing only. All animal procedures complied with the Institutional Animal Care and Use Committee guidelines and were approved by the Ethics Committee of Southern University of Science and Technology (Approval No. SUSTech‐JY202103018). Detailed pharmacologic rationale, compound selectivity, dosing parameters, and local exposure justification are provided in the Supplementary Methods and summarized in Table S2 (Supporting Information).

### Generation of Endothelial Conditional NOX2 Knockout Mice

*Cdh5*‐Cre and *Cybb* ^fl/fl^ mice were obtained from Cyagen Biosciences (Guangzhou, China). Endothelial‐specific *Nox2* knockout mice (EC‐*Nox2* ^−/−^) were generated by crossing *Cdh5*‐Cre with *Cybb* ^fl/fl^. Littermates carrying *Cybb* ^fl/fl^ but lacking Cre served as control (EC‐*Nox2* ^fl/fl^). All mice were on a C57BL/6 background and age‐matched male littermates were used for experiments. Genotyping was performed on tail DNA by PCR following the vendor's instructions. Deletion was further validated at the tissue level by qPCR and immunofluorescence in endothelial compartments as reported in the Results. All procedures were approved by the institutional animal ethics committee.

### Micro‐CT Evaluation

Peri‐implant bone was scanned on a SkyScan 1276 micro‐CT system (Bruker, Belgium) at 60 kV, 100 µA, 605 ms integration time, rotation step 0.4°, frame averaging 2, and pixel size 20.3 µm. A standardized peri‐implant volume of interest (VOI) was defined as a 200 µm concentric ring surrounding the threaded segment, excluding the cortical surface and metal–artifact region, and was applied uniformly across all samples. Reconstruction was performed using NRecon v1.7.4.6 (Bruker) with smoothing = 2, ring artifact correction = 6, and beam‐hardening correction = 40%. Thresholding and segmentation were conducted with fixed global grayscale thresholds (bone = 75–255; background = 0–74) using CTAn v1.20.8.0. Quantitative parameters, including tissue‐level bone mineral density (Ct.vBMD, mg·cm^−3^ and bone volume fraction (BV/TV, %), were extracted. 3D renderings were generated using CTVol v2.3.2.0, and representative examples of reconstructed peri‐implant regions are shown in Figure 1B to illustrate the VOI and segmentation workflow. All samples were analyzed using identical parameters to ensure reproducibility.

### Histomorphometric Analysis of Bone Healing Around Implants

Femora were fixed in 4% paraformaldehyde, decalcified in EDTA, paraffin‐embedded, and sectioned at 7 µm. Sections were stained with H&E, Masson's trichrome, and Van Gieson. ImageJ was used to quantify the peri‐implant bone area fraction and collagen‐positive fraction within a defined ROI adjacent to the implant track.

### Immunofluorescent Histochemistry at the BII

Fresh femora were cryo‐embedded in a gelatin medium and sectioned at 40 µm as described.^[^ ^53^, ^54^ ^]^ Sections were stained for CD31, EMCN, OCN, OSX, NOX2 with DAPI counterstaining. Confocal z‐stacks were acquired and rendered as maximum‐intensity projections and 3D surfaces. Quantification used a standardized peri‐implant ROI defined as a ring concentric with the screw (thread/metal‐artifact zone excluded). Within the ROI, CD31^+^EMCN^+^ (type H) endothelium and NOX2^+^/OSX^+^/OCN^+^ signals were quantified with identical thresholds across groups (Imaris).

### Flow Cytometry

After screw removal, bones were enzymatically dissociated to single‐cell suspensions following published protocols.^[^ ^8^, ^14^ ^]^ Live, non‐hematopoietic endothelial cells were identified as DAPI^−^CD45^−^Ter119^−^CD31^+^. Type H ECs were defined as CD31^+^EMCN^+^ within the CD31^+^ gate using FMO‐based thresholds. Data were acquired on a standard flow cytometer and analyzed in FlowJo.

For ROS measurements, cells were incubated with DCFH‐DA (10 µm), and DCF fluorescence was quantified in CD31^+^CD45^−^Ter119^−^ ECs.^[^ ^14^, ^55^ ^]^ In‐vitro ROS assays were performed analogously on cultured ECs.

### Quantitative Real‐Time PCR (qPCR)

Flow‐sorted endothelial cell (EC) fractions were lysed directly in RNA‐easy Isolation Reagent (Vazyme). cDNA synthesis and qPCR were performed as described.^[^ ^8^, ^14^ ^]^ In ECs, transcripts of coupling factors and *Nox* isoforms were quantified: *Nog*, *Vegfa*, *Fgf1*, *Pdgfa*, *Wnt3a*, *Bmp2*, and *Nox1–4*. In whole‐bone homogenates (freshly snap‐frozen and pulverized), endothelial/osteogenic markers were assessed: *Pecam1* (CD31), *Emcn*, *Runx2*, *Sp7* (Osterix), *Alp*, OPN, *Bglap*, and *Col1a1*. Expression was normalized to *Actb* using the ΔΔCt method. Primer sequences are provided in Table S3 (Supporting Information).

### Western Blot Analysis

Proteins from peri‐implant bone were extracted, quantified (BCA), separated by SDS‐PAGE, and transferred to PVDF (0.45 µm). Membranes were blocked (5% skim milk), incubated with primary antibodies (NOX2, GAPDH) overnight at 4 °C, followed by HRP‐conjugated secondary antibodies and ECL detection. Band intensities were quantified in ImageJ and normalized to GAPDH.

### Oxidative Stress Assays

Indices of oxidative stress in peri‐implant bone tissue homogenates were measured using colorimetric kits (Abbkine) according to the manufacturers’ instructions: lipid peroxidation, MDA, CAT activity, and SOD activity.

### Cell Culture and Transwell Co‐Culture

bEnd.3 and MC3T3‐E1 cells (Procell, Wuhan, China) were maintained in Dulbecco's Modified Eagle Medium (DMEM) supplemented with 10% fetal bovine serum and 1% penicillin–streptomycin at 37 °C in a humidified 5% CO_2_ atmosphere.

Normal‐glucose (NM) and high‐glucose (DM) media were defined as 5.5 and 25 mM glucose, respectively. In DM, 0.5 mm palmitate conjugated to bovine serum albumin (palmitate–BSA) was added to mimic diabetic metabolic stress, whereas the NM group received an equivalent BSA vehicle control.^[^ ^56^ ^]^ Exposure durations were consistent with the corresponding in vitro assays.

For osteogenic induction, MC3T3‐E1 cells were cultured in commercial osteogenic differentiation medium (Cyagen, Cat# MUXMT90021) with renewal every 3 days. Cells at passages 4–7 were seeded onto mechanically polished Ti6Al4V disks in 24‐well plates at a density of 1 × 10⁴ cells mL^−1^ and allocated to NM or DM conditions; pharmacologic agents (TMF, GSK2795039, NAC) were added as specified in the experimental design.

For the Transwell co‐culture systerm, bEnd.3 cells were seeded in the lower chamber and MC3T3‐E1 cells on Ti disks placed in were 24‐well Transwell inserts (0.4 µm pores) under NM or DM conditions.

### In Vitro Functional Assays

Unless noted, assays were performed on Ti disks.

### Cell Viability (CCK‐8)

Proliferation of bEnd.3 and MC3T3‐E1 on Ti was assessed at day 4 using 1:10 CCK‐8: medium mixture (100 µL per well, 1–4 h incubation); absorbance was read at 450 nm.

### Apoptosis (Flow Cytometry)

On day 7, cells were stained with Annexin V–FITC/propidium iodide (PI) for 30 min after PBS washes**;** early/late apoptosis was determined by flow cytometry and analyzed in FlowJo.

### Cell Adhesion

After 3 days on Ti, bEnd.3 and MC3T3‐E1 were fixed (4% PAF), stained for vinculin (focal adhesions), and counterstained with phalloidin (F‐actin) and DAPI. Fluorescence images were acquired by confocal microscopy; vinculin signal and cell spreading were quantified as indicated in the Results.

### Osteogenic Differentiation and Mineralization

MC3T3‐E1 cells were cultured in osteogenic induction medium. ALP was assessed on day 14 by colorimetric staining and by enzymatic activity quantification at 405 nm. Mineralization was evaluated on day 21 by ARS (2% w/v, pH 4.2) staining; bound dye was extracted with 10% acetic acid (overnight), neutralized with 10% ammonium hydroxide**;** read at 405 nm.

### Immunofluorescence Staining (Osteogenic/Angiogenic Markers)

At the indicated time points (e.g., RUNX2/VEGFA on day 3; OCN on day 28), cells were fixed, permeabilized, blocked, and probed overnight with primary antibodies against OCN, RUNX2, and VEGFA. Following PBS washes, Alexa Fluor–conjugated secondary antibodies were applied. Images were acquired by confocal microscopy (Zeiss LSM 980) using identical settings across groups.

### Tube Formation Test (In Vitro Angiogenesis)

bEnd.3 previously cultured on Ti (7 days) were detached, resuspended in 1% FBS medium, and plated on growth factor–reduced Matrigel in 96‐well plates (2–3 × 10⁴ cells per well). Tube formation was imaged at 3–6 h. Branch points and cumulative tube length were quantified from phalloidin‐labeled images using ImageJ.

### Wound‐Healing Migration Assay

Confluent bEnd.3 monolayers were serum‐reduced, scratched with a pipette tip, and rinsed to remove detached cells. Wound areas were imaged at 0, 6, 12, 18, and 24 h at the same positions and quantified in ImageJ as the percentage of wound closure.

### CRISPR/Cas9‐Medicated *Cybb* Knockout in bEnd3

A high‐efficiency 20‐nt sgRNA targeting *Cybb* (forward 5′‐CACCGAACTCAGAATCCGGCCCGCG‐3′; reverse 5′‐AAACCGCGGGCCGGATTCTGAGTTC‐3′) was cloned into PX458 (pSpCas9(BB)‐2A‐GFP). bEnd.3 cells were transfected with PX458‐sgRNA (Lipofectamine 3000). At 24 h, GFP⁺ cells were sorted, single‐cell–plated into 96‐well plates, and expanded. *Cybb* disruption was verified by genomic PCR/TA‐cloning/Sanger sequencing; KO efficiency was confirmed by NOX2 Western blot (Figure S7, Supporting Information).

### RNA Sequencing and Bioinformatics

Total RNA was isolated from Ti co‐cultures under diabetic conditions (bEnd.3: EC‐KO, EC‐WT; MC3T3‐E1: OB‐KO, OB‐WT; *n* = 3 per group). rRNA was depleted, RNA fragmented to ≈200 nt, and strand‐specific libraries were prepared per manufacturer's instructions;^[^ ^57^ ^]^ libraries were QC‐checked and sequenced by Wuhan Kang test Technology Co., Ltd. (China). Raw reads underwent QC and alignment to the mouse reference genome using TopHat2; gene‐level counts were obtained and FPKM values computed for correlation/visualization. PCA and sample‐to‐sample correlations assessed replicate reproducibility. Differential expression was evaluated within cell types (EC: KO vs WT; OB: KO vs WT) with thresholds |log_2_FC**| >** 1 and q < 0.001 (Benjamini–Hochberg). Functional enrichment of DEGs used GO and KEGG; where indicated, GSEA interrogated EndMT and inflammatory/fibrotic pathways.

### Statistical Analyses

Statistical analyses were performed using GraphPad Prism v9.0 (GraphPad Software, San Diego, CA, USA). For normally distributed data, unpaired two‐tailed Student's *t*‐tests were used for comparisons between two groups, and one‐way ANOVA with Tukey's post hoc test was applied for comparisons involving three or more groups. For non‐normal data, the Mann–Whitney U test (two groups) or Kruskal–Wallis test with Dunn's multiple comparisons (≥3 groups) was used. For longitudinal data, two‐way ANOVA with appropriate post‐hoc tests was performed. Data were presented as mean ± SD. Sample size (n) is indicated in the figure legends. Statistical significance was indicated as: P < 0.05 (*), P < 0.01 (**), P < 0.001 (***), P < 0.0001 (****); ns, not significant. For RNA‐seq analyses, multiple testing correction was applied using the Benjamini–Hochberg false discovery rate (FDR) with thresholds of |log_2_FC| > 1 and q < 0.001.

## Conflict of Interest

The authors declare no conflict of interest.

## Author Contributions

Z.M.W., Q.D.H., and Y.L. contributed equally to this work. Z.M.W. performed validation, investigation, formal analysis, wrote the original draft, and visualization. Q.D.H. performed validation, investigation, and formal analysis. Y.L. and T.T.C. performed validation and investigation. K.K.Y. and L.Y.L. performed investigation and formal analysis. L.W. performed conceptualization, investigation, wrote, reviewed, and edited the draft, visualization, supervision, project administration, and funding acquisition.

## Supporting information



Supporting Information

## Data Availability

The data that support the findings of this study are available from the corresponding author upon reasonable request.
