# Supplementary material for: NOX2‐Driven Oxidative Stress Promotes EndMT and Uncouples Angiogenesis–Osteogenesis at the Bone–Implant Interface in Diabetes
Source: Adv Sci (Weinh). 2025 Nov 10;13(4):e17885. doi: 10.1002/advs.202517885 (PMC12822455; doi:10.1002/advs.202517885)
Supplement: Supplementary file 1 — Supporting Information [file ADVS-13-e17885-s001.docx]

**Supporting Information**

**NOX2-Driven Oxidative Stress Promotes EndMT and Uncouples Angiogenesis–Osteogenesis at the Bone–Implant Interface in Diabetes**

Zimei Wu, Qiaodan Hou, Yang Liu, Tingting Chen, Kunkun Yang, Luyao Li, Lin Wang*

Z. Wu, Q. Hou, T. Chen, K. Yang, L. Li, L. Wang

Department of Biochemistry, SUSTech Homeostatic Medicine Institute, School of Medicine, Southern University of Science and Technology, Shenzhen 518055, Guangdong, China

Email: wangl7@sustech.edu.cn

Z. Wu

Emergency and Disaster Medical Center, The Seventh Affiliated Hospital, Sun Yat-Sen University, Shenzhen, 518107, China

Y. Liu

Department of Biomedical Engineering, Southern University of Science and Technology, Nanshan District, Shenzhen, 518055, P. R. China.

**Supplemental Figures:**


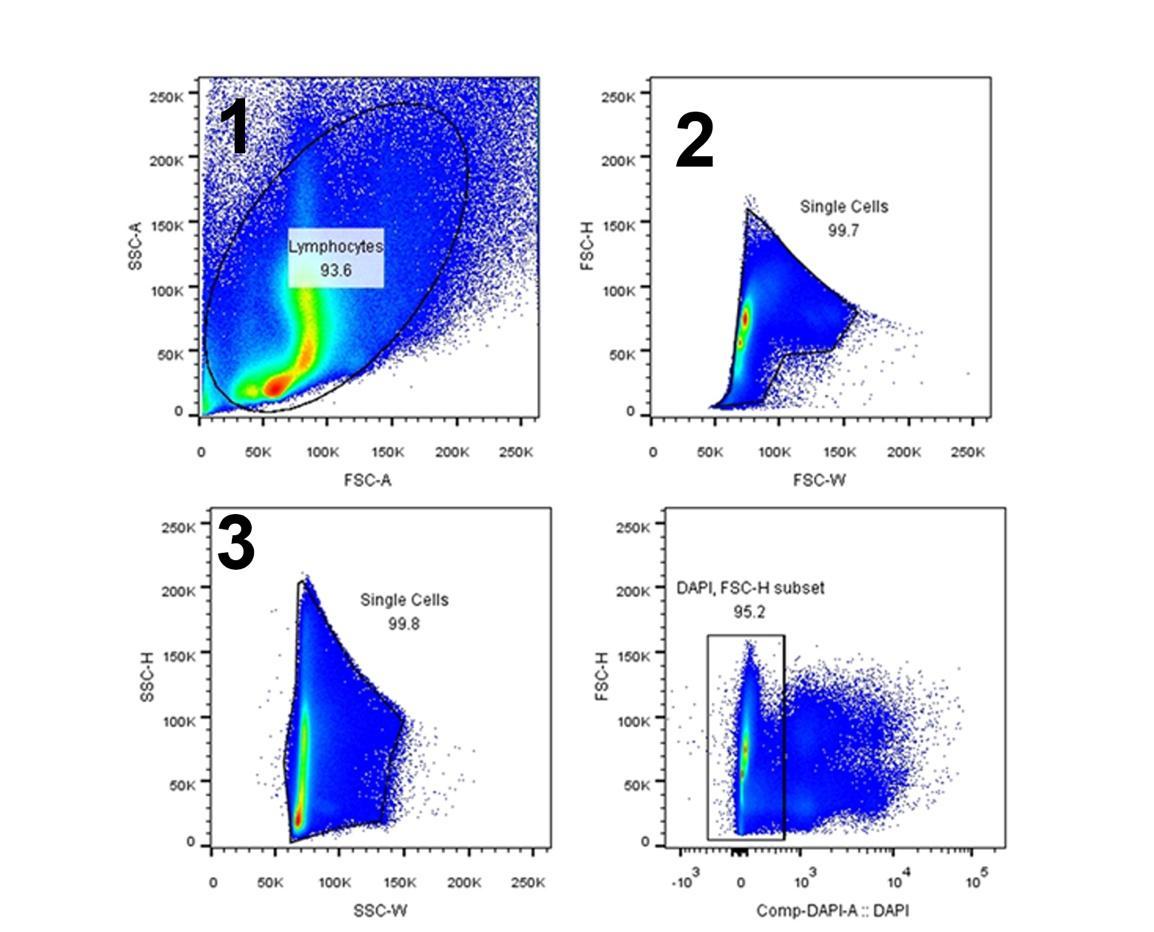


**Figure S1**

**Pre-gating strategy for flow cytometry. Representative gating used for all flow experiments on peri-implant bone digests.** (1) FSC-A vs SSC-A gate to select cells and exclude debris (size/granularity). (2) FSC-H vs FSC-W and (3) SSC-H vs SSC-W gates to remove doublets and retain single cells. (4) DAPI vs FSC-H gate to exclude non-viable events (DAPI⁺) and yield live, single cells for downstream analysis. Percentages indicate the fraction of events retained at each step. These pre-gates were applied prior to antibody-based identification of subsets (e.g., CD31⁺EMCN⁺ type-H ECs with CD45/Ter119 exclusion) in the main and supplementary figures.


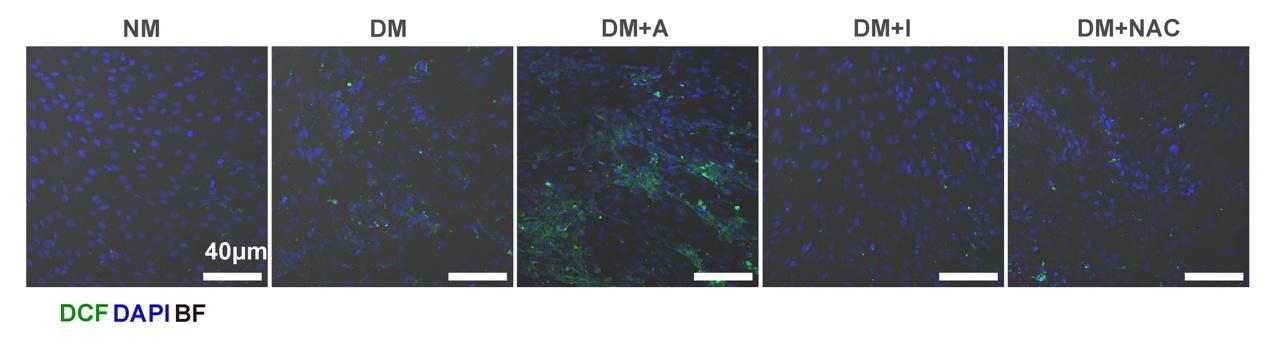


**Figure S2**

**bEnd.3 ROS imaging (DCF) on titanium under diabetic conditions.** Representative fields of bEnd.3 cultured on Ti under the indicated conditions: NM, DM, DM+A (TMF, NOX2 agonist), DM+I (GSK2795039, NOX2 inhibitor), and DM+NAC (ROS scavenger). DCF (green) reports intracellular ROS; nuclei are counterstained with DAPI (blue); bright-field (BF) is overlaid for context. A diffuse increase in DCF fluorescence is evident in DM, which is further enhanced by DM+A and attenuated by DM+I and DM+NAC, consistent with pharmacologic modulation of NOX2. Scale bar, 40 μm.


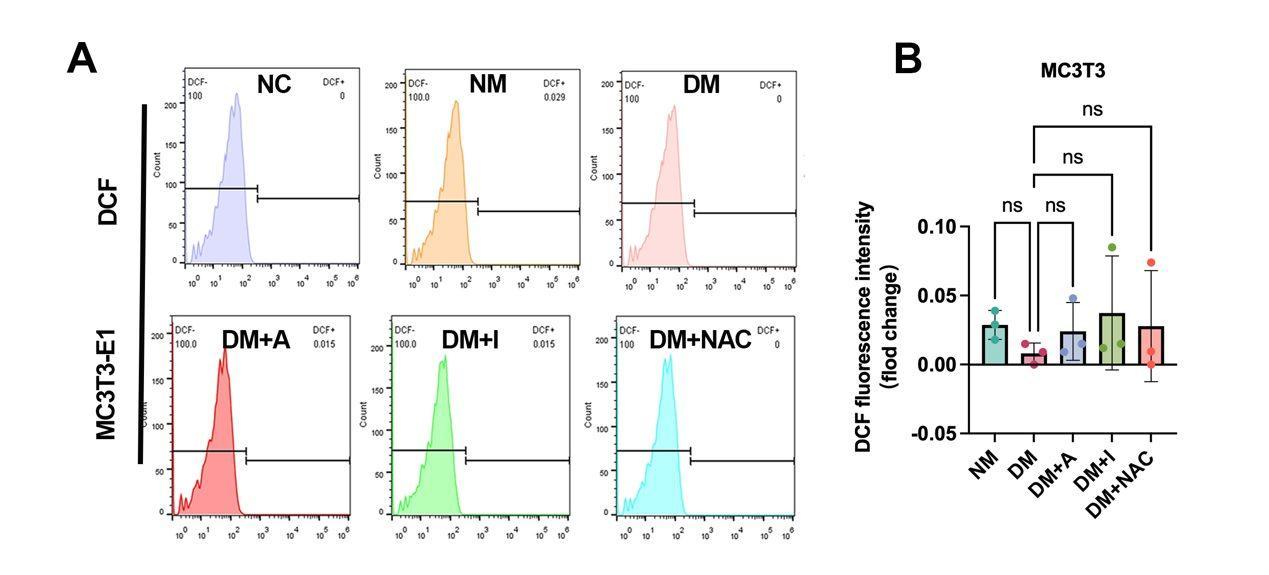


**Figure S3**

**DCF flow cytometry of MC3T3 on Ti under NM, DM, DM+A (TMF), DM+I (GSK2795039), and DM+NAC.** (A) Representative DCF histograms with gate. (B) Quantification of DCF fluorescence intensity (fold change vs. NM). No significant differences across groups (one-way ANOVA with Tukey; n=3).


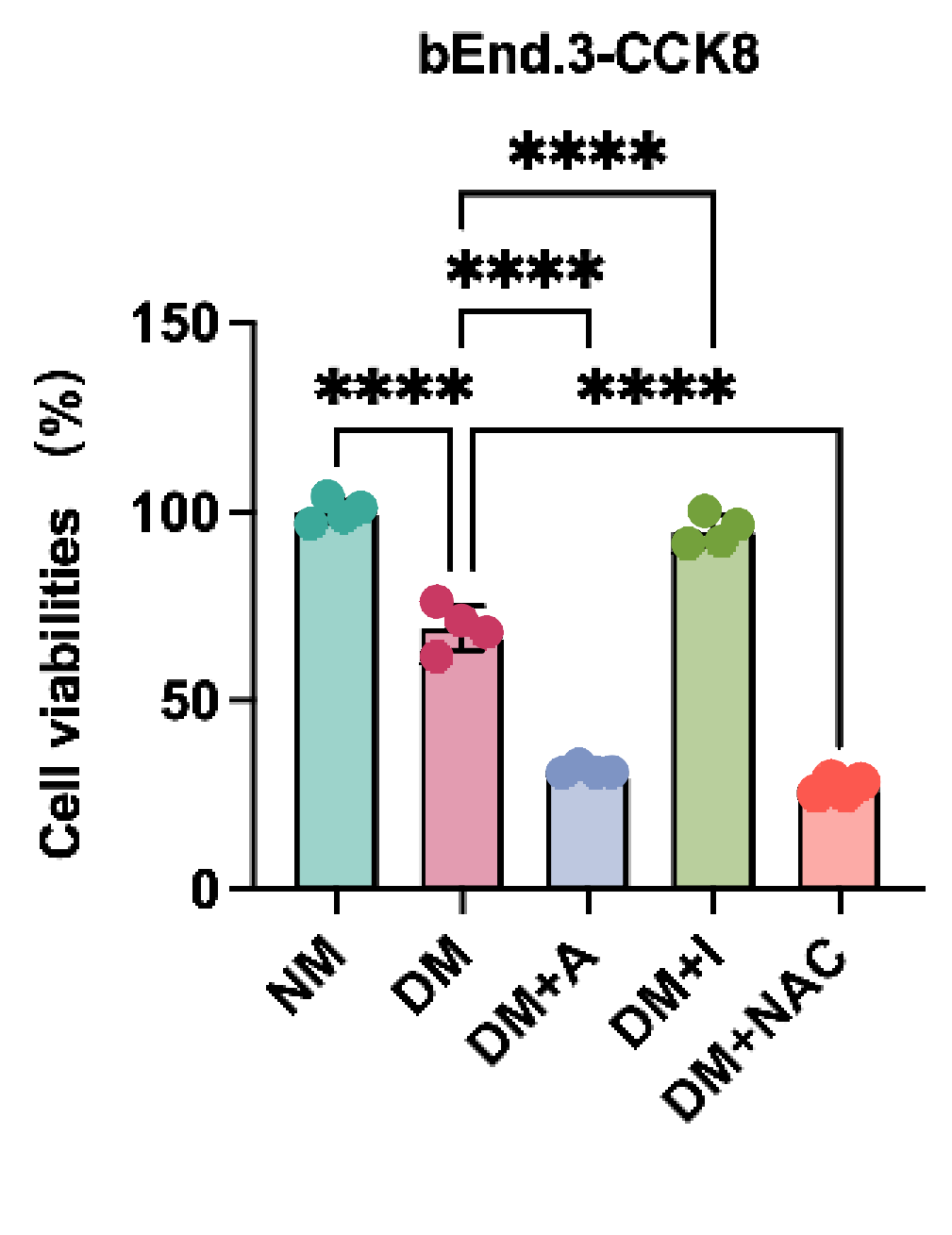


**Figure S4**

**Endothelial viability on titanium under diabetic conditions.** CCK-8 assays of bEnd.3 cultured on Ti in NM, DM, DM+A (TMF, NOX2 agonist), DM+I (GSK2795039, NOX2 inhibitor), and DM+NAC (ROS scavenger). Bars show mean ± SD percent viability normalized to NM (=100%); dots represent biological replicates. One-way ANOVA with Tukey’s post-hoc test; P < 0.05, P < 0.01, P < 0.001, P < 0.0001.


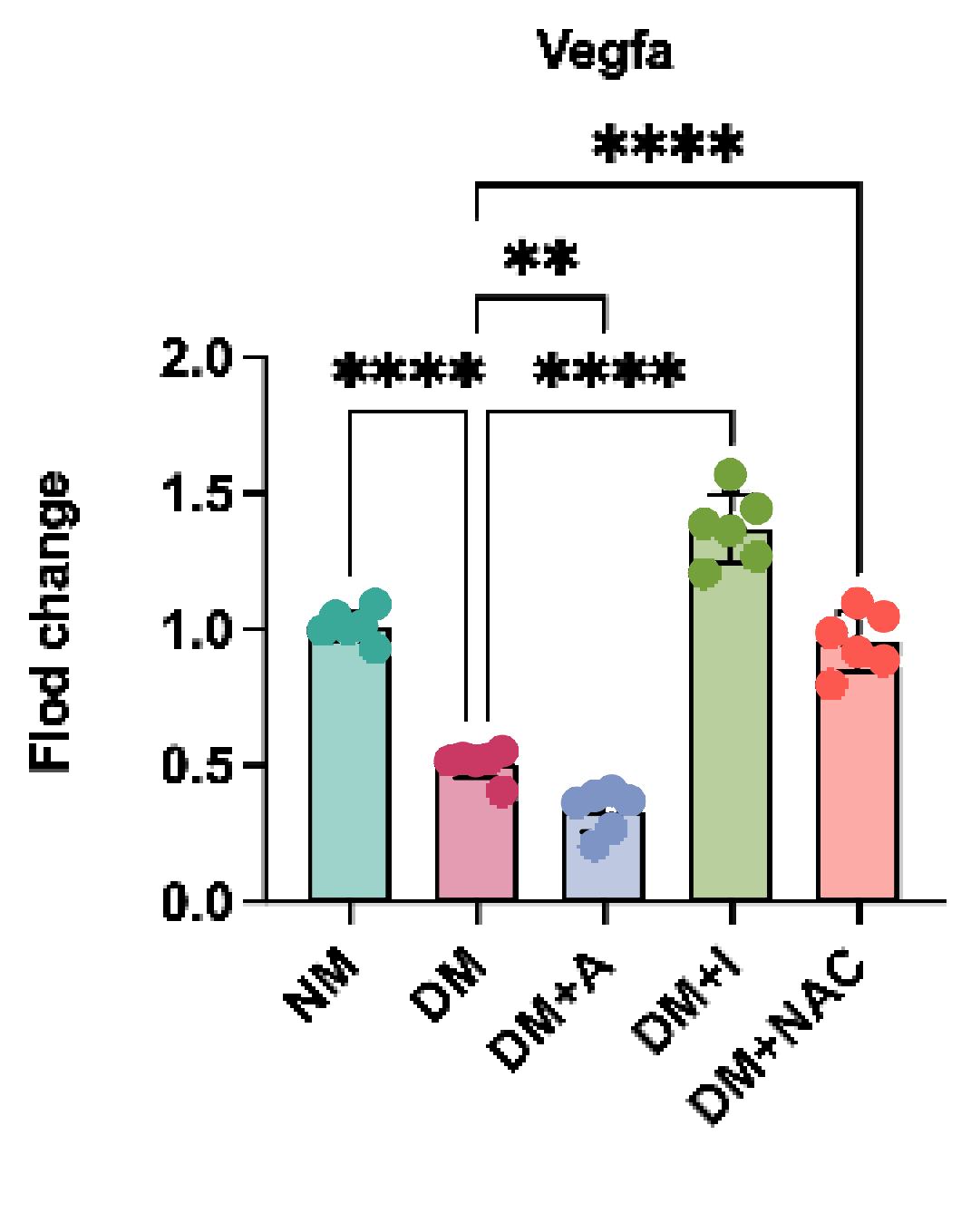


**Figure S5**

**Vegfa transcription in bEnd.3 on titanium under diabetic conditions.** qPCR analysis of Vegfa in bEnd.3 cultured on Ti under NM, DM, DM+A (TMF), DM+I (GSK2795039), and DM+NAC. Expression was normalized to the housekeeping control (ΔΔCt) and expressed relative to NM (=1.0). Bars show mean ± SD; dots denote biological replicates. One-way ANOVA with Tukey’s post-hoc test; P < 0.05, P < 0.01, P < 0.001, P < 0.0001.
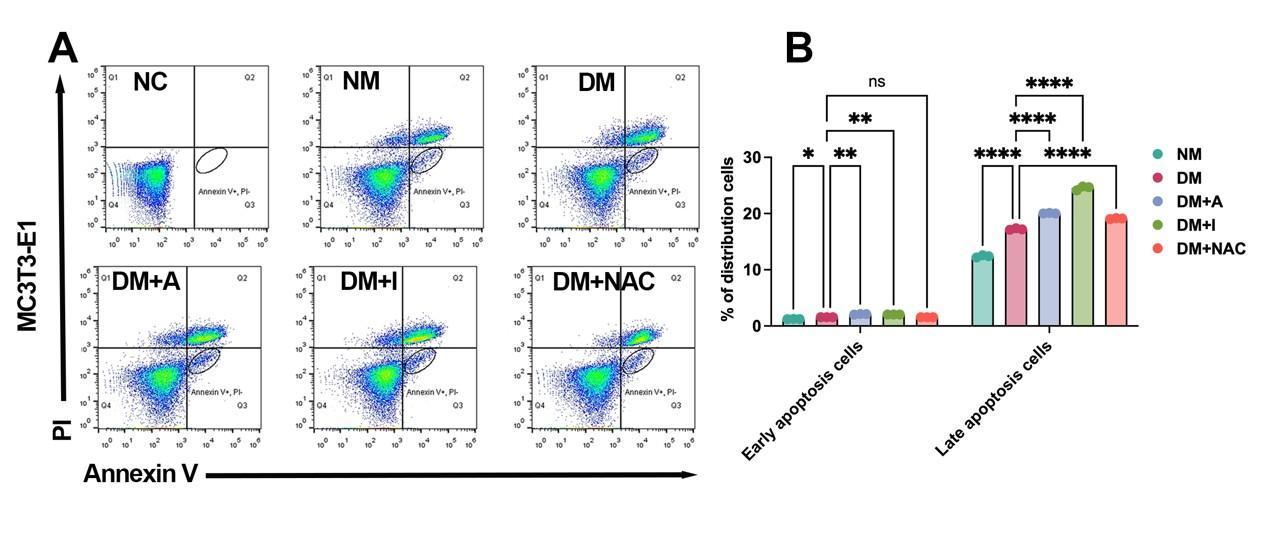


**Figure S6**

**Apoptosis of MC3T3 monocultures on titanium under diabetic conditions.** (A) Representative Annexin V/PI plots for NM, DM, DM+A (TMF, NOX2 agonist), DM+I (GSK2795039, NOX2 inhibitor), and DM+NAC. (B) Quantification of early and late apoptotic fractions. Late apoptosis is higher in DM vs NM, further increased by TMF, and not reduced by GSK2795039; NAC shows no significant decrease versus DM. Early apoptosis shows no significant differences among groups. n=3; one-way ANOVA with Tukey; ns, not significant; P<0.05; P<0.01; P<0.001; P<0.0001.


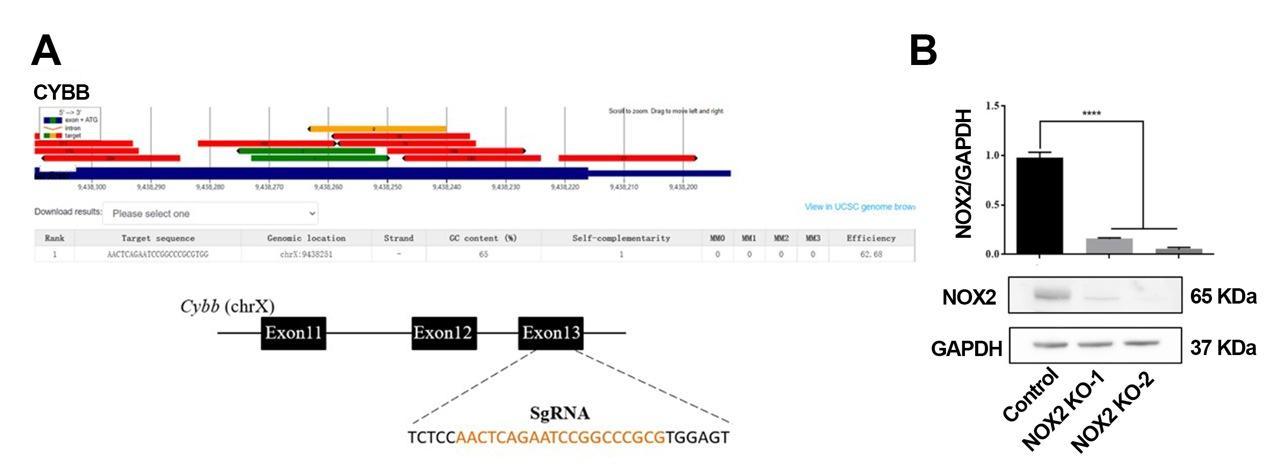


**Figure S7**

**CRISPR/Cas9 design and validation of NOX2 knockout in endothelial cells.** (A) sgRNA design for the mouse Cybb (NOX2) locus (chrX). Tracks show candidate guides across exons; the selected guide (shown with its PAM in the panel) targets the coding region between Exon 11–13. (B) Immunoblot of NOX2 with GAPDH loading control in parental bEnd.3 (Control) and two independent knockout clones (NOX2 KO-1, NOX2 KO-2). Densitometry (NOX2/GAPDH, normalized to Control = 1) demonstrates near-complete loss of NOX2 protein in both KO clones. Data are mean ± SD (n = 3 independent experiments). One-way ANOVA with Tukey’s post-hoc test; ****P < 0.0001.


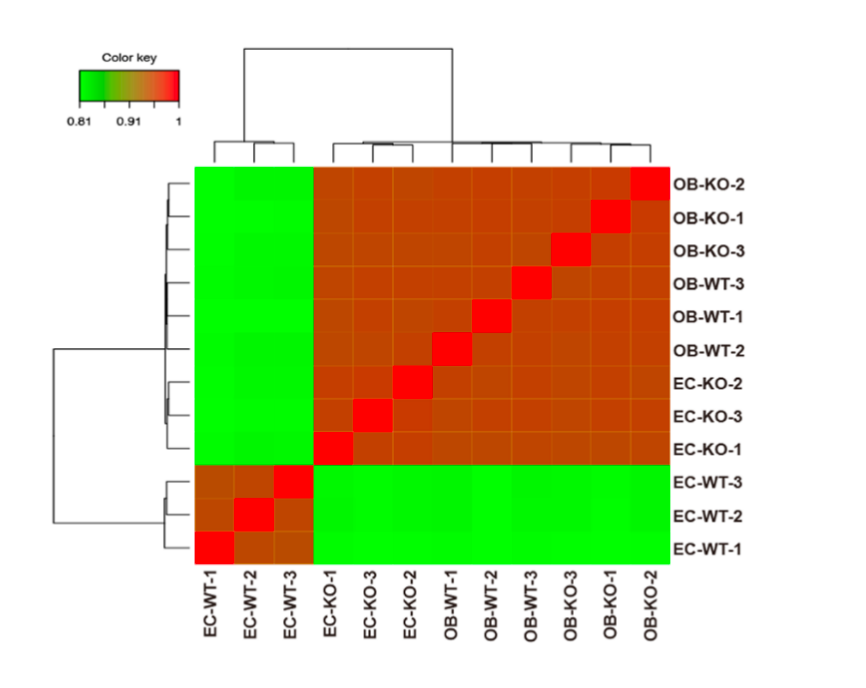
**Figure S8**

**RNA-seq quality control.** Sample-to-sample correlation heatmap with hierarchical clustering for all EC and OB libraries (n = 3 per condition). Replicates cluster tightly within groups and separate by compartment and genotype (EC-WT vs EC-KO; OB-WT vs OB-KO).


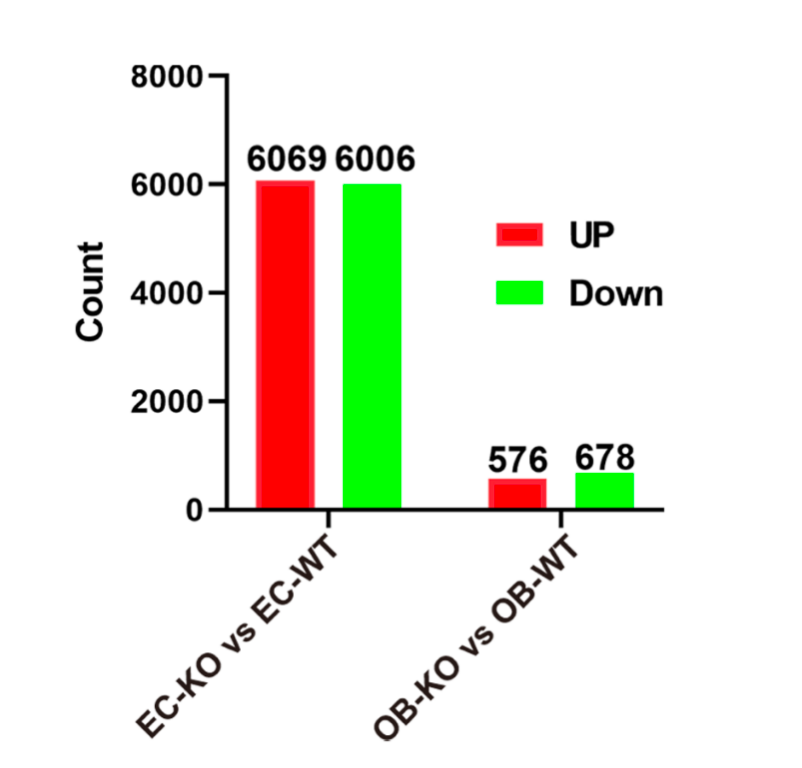


**Figure S9**

**RNA-seq DEG overview.** Bar chart of differentially expressed genes (DEGs) for EC-KO vs EC-WT and OB-KO vs OB-WT (|log₂FC| > 1; Benjamini–Hochberg q < 0.001), showing a dominant endothelial response and a moderate osteoblast response consistent with paracrine remodeling.


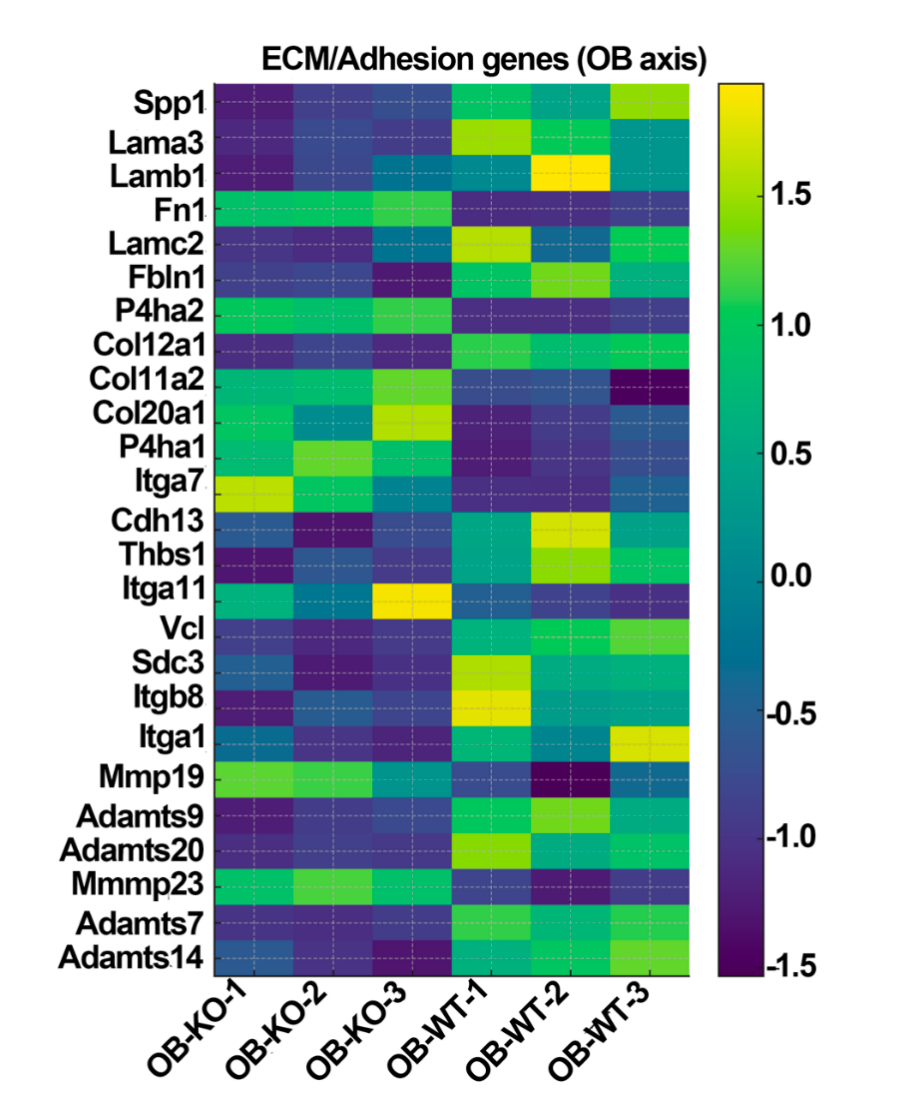


**Figure S10**

**OB-axis ECM/adhesion gene set.** Heatmap of selected extracellular-matrix and adhesion-related genes in osteoblasts (OB-WT vs OB-KO), z-scored per gene. Notable decreases in Fn1, laminins (Lama3/Lamb1/Lamc2), collagens (Col11a2/Col12a1/Col20a1), integrins (Itga1/7/11, Itgb8), and Adamts family members are evident in OB-KO, consistent with attenuated adhesion/guidance programs.


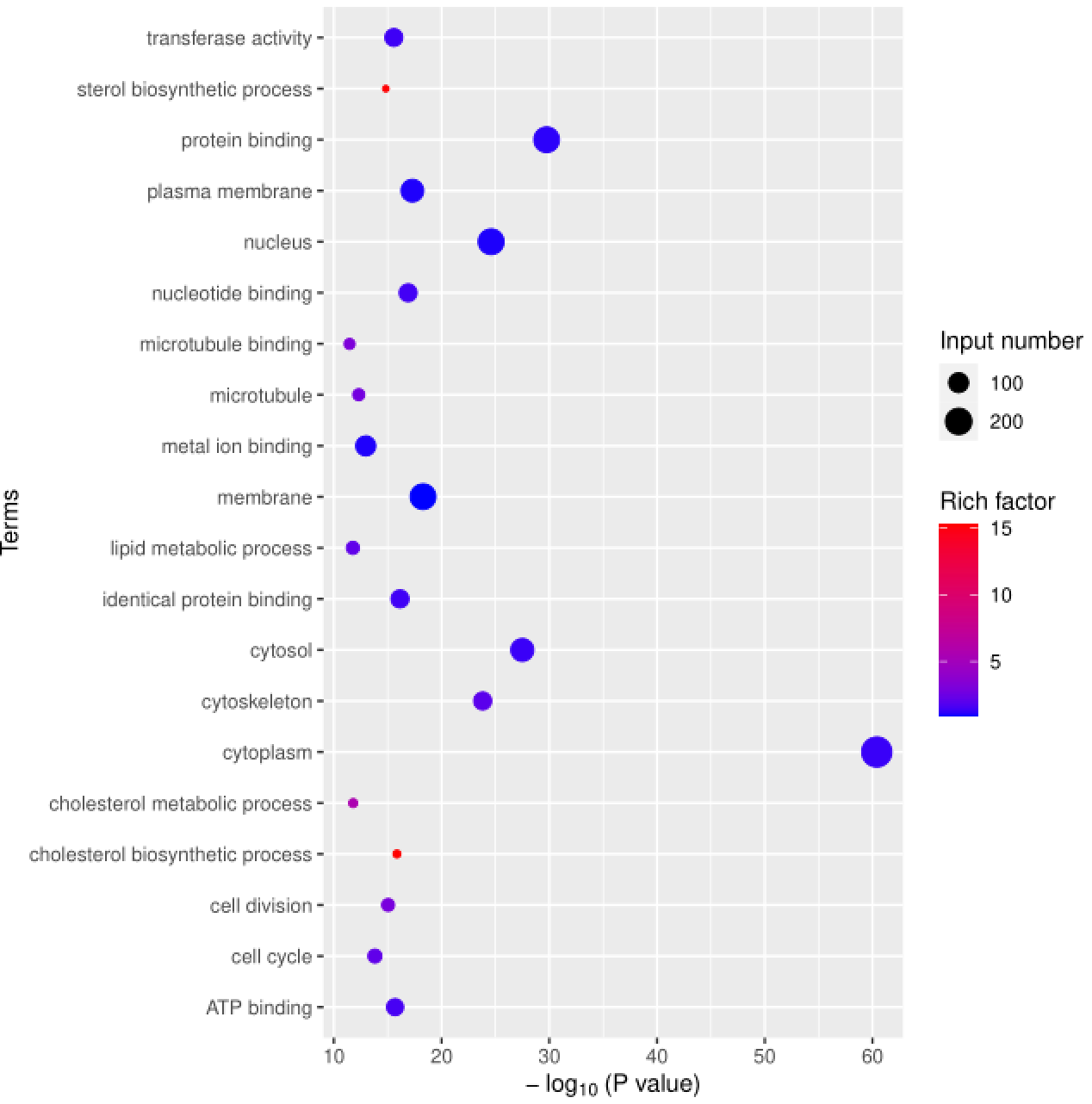


**Figure S11**

**GO enrichment for OB DEGs.** GO terms enriched among down-regulated OB genes (OB-KO vs OB-WT) across BP/CC/MF, highlighting membrane/cytoskeleton components and lipid/sterol metabolic processes. Dot size indicates gene count; color encodes rich factor; x-axis, −log₁₀(P value).


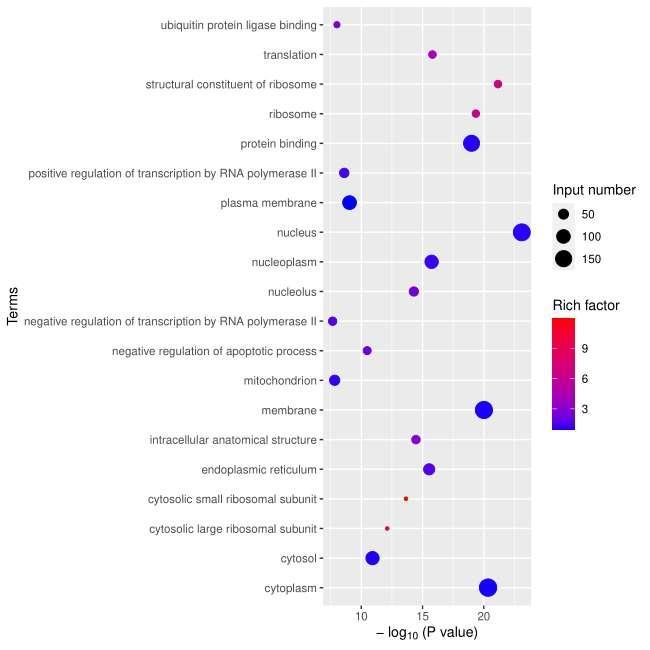


**Figure S12**

**GO enrichment for OB DEGs.** GO terms enriched among up-regulated OB genes, including cytoplasm/nucleus/ER/ribosomal constituents and processes related to translation, transcriptional regulation, and apoptosis control. Dot size indicates gene count; color encodes rich factor; x-axis, −log₁₀(P value).


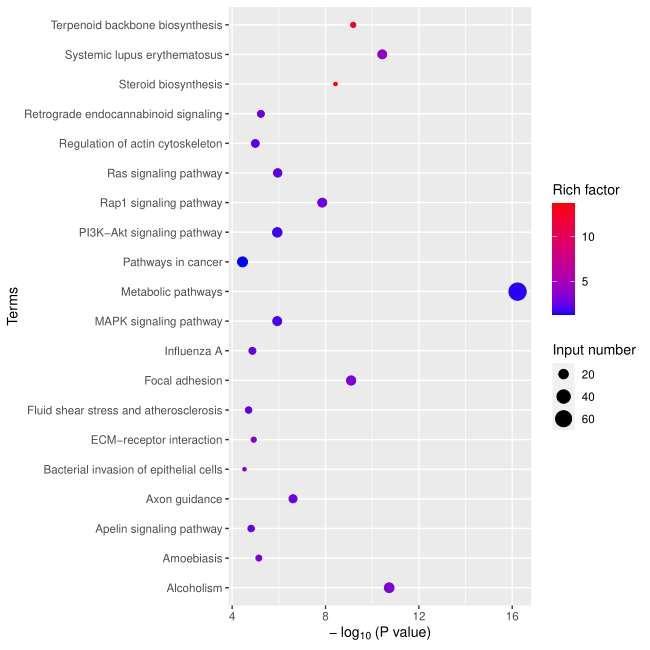


**Figure S13**

**KEGG enrichment for OB DEGs.** KEGG pathways enriched among down-regulated OB genes (e.g., ECM–receptor interaction, focal adhesion, regulation of actin cytoskeleton, PI3K–AKT, MAPK, axon guidance). Dot size indicates gene count; color encodes rich factor; x-axis, −log₁₀(P value). Multiple testing controlled by BH FDR (q < 0.001 threshold for gene selection).


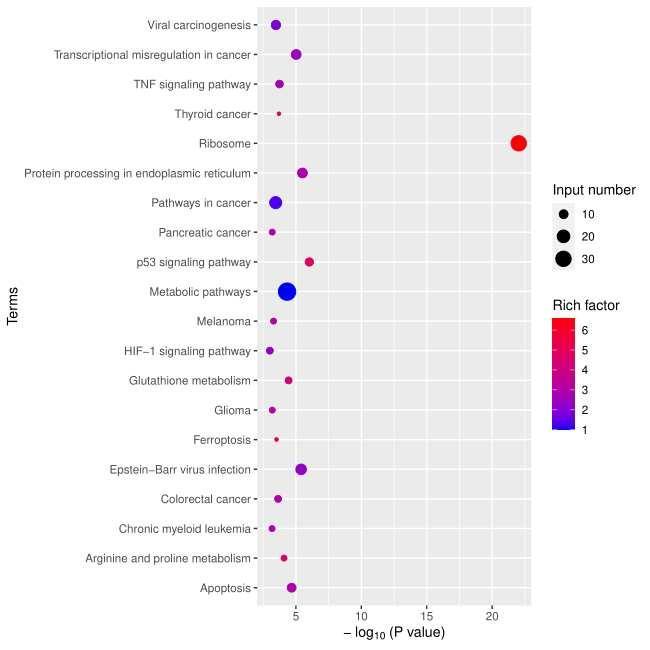


**Figure S14**

**KEGG enrichment for OB DEGs.** Pathways enriched among up-regulated OB genes (e.g., ribosome, protein processing in endoplasmic reticulum, and selected metabolic/stress-response pathways). Dot size indicates gene count; color encodes rich factor; x-axis, −log₁₀(P value). Multiple testing controlled by BH FDR (q < 0.001 threshold for gene selection).


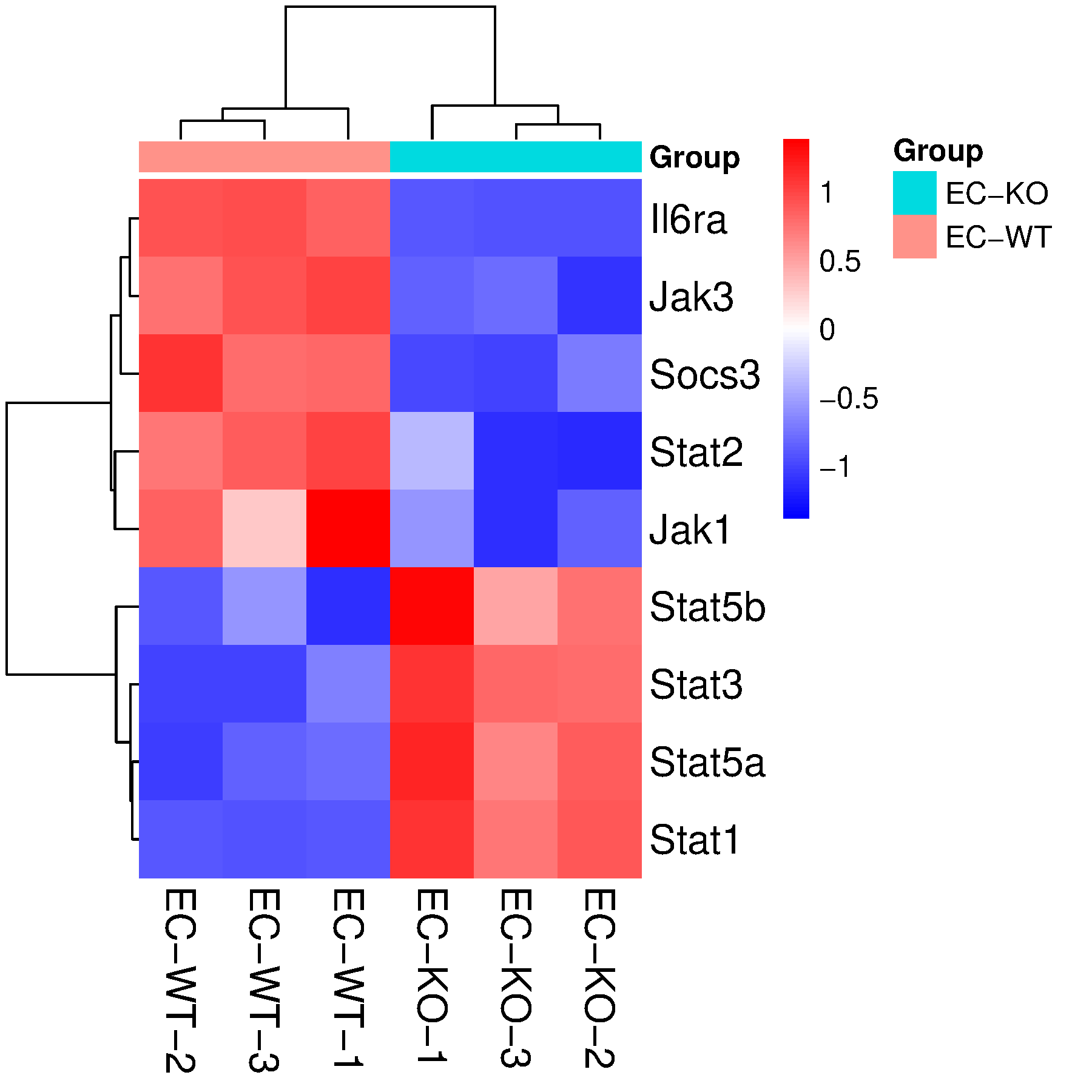


**Figure S15**

**Expression heatmap of representative JAK–STAT signaling genes in endothelial cells (EC-KO vs EC-WT).** The heatmap shows normalized RNA-seq expression values (z-scored per gene) for key components of the JAK–STAT axis (JAK1, JAK3, STAT1, STAT2, STAT3, STAT5A, STAT5B, SOCS3, IL6RA). Each column represents a biological replicate of EC-WT or EC-KO samples (n = 3 per group). Lower expression levels (blue) indicate transcriptional down-regulation of cytokine-responsive genes in EC-KO cells relative to EC-WT, supporting suppression of the JAK–STAT-associated inflammatory axis upon NOX2 deletion. Color scale represents row-scaled z-scores of log₂-transformed normalized counts.

**Table S1**

**Selected JAK–STAT-axis genes identified from endothelial RNA-seq (EC-KO vs EC-WT).**

| Gene | log₂FC (EC-KO vs EC-WT) | P value |
| --- | --- | --- |
| Jak1 | -0.42 | 3.12 × 10⁻⁷ |
| Jak3 | -2.35 | 8.79 × 10⁻^9^ |
| Stat1 | 1.20 | 1.14 × 10⁻^9^ |
| Stat2 | -0.51 | 7.96 × 10⁻⁷ |
| Stat3 | 0.77 | 1.24 × 10⁻^6^ |
| Stat5a | 0.83 | 5.34 × 10⁻^8^ |
| Stat5b | 0.37 | 2.73 × 10⁻5 |
| Socs3 | -1.00 | 2.45 × 10⁻^8^ |
| Il6ra | -4.80 | 2.94 × 10⁻^14^ |

**Supplemental Methods:**

**Pharmacologic agents and dosing rationale**

GSK2795039 (C₁₆H₁₃NO₃; MedChemExpress) is a small-molecule, cell-permeable NOX2 inhibitor that competitively interferes with the NADPH-binding site of the gp91^phox catalytic subunit. It has been characterized as a selective NOX2 inhibitor with demonstrated in-vivo activity in rodent models of vascular and neuro-inflammation, showing minimal cross-reactivity with other NOX isoforms at effective concentrations ^1, 2^.

4′,6,7-Trimethoxyisoflavone (TMF; C₁₈H₁₆O₅; MedChemExpress) is a naturally occurring isoflavone reported to induce NOX2-dependent ROS generation through a p47^phox-dependent mechanism in keratinocytes, thereby enhancing oxidative and migratory signaling ^3^.

All pharmacologic agents were freshly prepared in sterile saline at 4 mM concentration. Ten microliters of each solution were injected periosteally at the distal femur every 3 days for 2 weeks, beginning on the day of implantation. NM and DM control groups received saline.

This localized periosteal injection strategy confines exposure to the bone–implant interface (~1 mm diffusion radius), ensuring sufficient local concentration while minimizing systemic spillover. The plausibility of high local exposure is supported by prior studies demonstrating effective peri-implant drug delivery, such as N-acetylcysteine-loaded titanium nanotube implants ^4^ and titanium-based local drug-delivery systems ^5^.Dosing parameters for all agents are summarized in Supplementary Table S2.

**Table S2**

**Summary of pharmacologic dosing parameters used in vivo**

| Agent | Function | Conc. | Volume | Route | Schedule | Vehicle/  Control | Key refs |
| --- | --- | --- | --- | --- | --- | --- | --- |
| **GSK2795039** | NOX2 inhibitor | 4 mM | 10 µL | Periosteal (distal femur) | q3d × 2 wk | Saline (DM vehicle) | [1-2] |
| **TMF (4′,6,7-Trimethoxyisoflavone)** | Reported NOX2 activator | 4 mM | 10 µL | Periosteal | q3d × 2 wk | Saline (DM vehicle) | [3] |
| **NAC (N-acetylcysteine)** | ROS scavenger | 4 mM | 10 µL | Periosteal | q3d × 2 wk | Saline (DM vehicle) | [4-5] |
| **Saline** | Vehicle control | — | 10 µL | Periosteal | q3d × 2 wk | — | — |

**Table S3**

**Primers used in qPCR**

| Gene | Forward primer sequence (5’-3’) | Reverse primer sequence (3’-5’) |
| --- | --- | --- |
| ITGB3 | GGAAGGCTGGCAGGCATTGTC | ATGGTAGTGGAGGCAGAGTAGTGG |
| CoL18a1 | ATCCCTACAGTCCTCCCCTCTCC | CAGCGTGCCAGCCTCAGTTG |
| SIRT1 | CCAGACCTCCCAGACCCTCAAG | GTGACACAGAGACGGCTGGAAC |
| SOD2 | TCCCAGACCTGCCTTACGACTATG | CTCCTCGGTGGCGTTGAGATTG |
| CAT | GGAGGCGGGAACCCAATAGGAG | TGTCAAAGTGTGCCATCTCGTCAG |
| NOX1 | CTGGAACAAGAGATGGAGGAATTAGGC | GTCAGTGGCTCTGTCAAAGTTTAATGC |
| NOX2 | GAAGACAACTGGACAGGAACCTCAC | AAATCCCGACTCTGGCATTCACAC |
| Runx2 | TCCCAGGCAGGCACAGTCTTC | AGCGGCGTGGTGGAGTGG |
| ALP | CACGGCGTCCATGAGCAGAAC | CAGGCACAGTGGTCAAGGTTGG |
| BMP-2 | GCCAAACACAAACAGCGGAAGC | GGTGCCACGATCCAGTCATTCC |
| BGLAP2 | TGGCTGCGCTCTGTCTCTCTG | GGATCTGGGCTGGGGACTGAG |
| Col1a1 | GCTCAGAGGCGAAGGCAACAG | GATGGGCAGGCGGGAGGTC |
| EMCN | GACCCAGGCACTCCAGAAAACG | CTGTGCAGAGTGTTCGCCAGAC |
| Pecam1 | ACCCTGGAGTGCCTTGTGGAC | AGACCCGAGCCTGAGGAATGAC |
| Noggin | CAGCACCCAGCGACAACCTG | CAGCAGCGTCTCGTTCAGATCC |
| Pdgfa | CAGCGTCAAGTGCCAGCCTTC | GGTCGCACATGCACACTCCAG |
| Tgfb1 | GGTTGCCAGCCAAGTGACATAGAG | ATCCCTTTGCTGCGATTGGTGAC |
| Fgf1 | CACAACCTTCGCAGCCCTGAC | TGGTCGCTCCTGTCCCTTGTC |
| Wnt3a | GGGTGTCAAAGCGGGCATCC | GGCTGTTGCTGACGGTGGTG |
| Bgalp | TGGCTGCGCTCTGTCTCTCTG | GGATCTGGGCTGGGGACTGAG |
| Ibsp | GGACTGCCGAAAGGAAGGTT | CCGGTACTTAAAGACCCCGTTT |
| FGF18 | TGTGCTTCCAGGTTCAGGTGTTG | GCTGCTTCCGACTCACATCATCTC |
| FGFr3 | GGTGGTCATGGCAGAAGCTATTGG | CAGGTCCTTGTCAGTCGCATCATC |
| FGF21 | GCACACCGCAGTCCAGAAAGTC | TGGCTGTTGGCAAAGAAACCTAGAG |
| ANGPTL6 | GTGTAGTAGCCGTGTGGTGTGAAC | TTGTAGTGCTGCCAGTTGGTGAAG |
| Pdgfa | TGAGATCGAAGGCAGGCACATTTAC | GCGGCAAGGTATGATGGCAGAG |
| Wnt9a | CAGCAGCAAGTTTGTCAAGGAGTTC | CCTTTATCACCTTCACACCCACGAG |
| Actb | GTGACGTTGACATCCGTAAAGA | GCCGGACTCATCGTACTCC |

**References**

[1] K. Hirano, W. S. Chen, A. L. W. Chueng, A. A. Dunne, T. Seredenina, A. Filippova, S. Ramachandran, A. Bridges, L. Chaudry, G. Pettman, C. Allan, S. Duncan, K. C. Lee, J. Lim, M. T. Ma, A. B. Ong, N. Y. Ye, S. Nasir, S. Mulyanidewi, C. C. Aw, P. P. Oon, S. Liao, D. Li, D. G. Johns, N. D. Miller, C. H. Davies, E. R. Browne, Y. Matsuoka, D. W. Chen, V. Jaquet,A. R. Rutter *Antioxidants & Redox Signaling* **2015**, 23, 358-374.

[2] M. G. de Oliveira, F. Z. Monica, G. R. Passos, J. A. Victorio, A. P. Davel, A. L. L. Oliveira, C. A. Parada, C. A. L. D'Ancona, W. G. Hill,E. Antunes *Antioxidants (Basel)* **2022**, 12,

[3] N. T. Bui, M. T. Ho, Y. M. Kim, Y. Lim,M. Cho *Phytomedicine* **2014**, 21, 570-7.

[4] Y. H. Lee, G. Bhattarai, I. S. Park, G. R. Kim, G. E. Kim, M. H. Lee,H. K. Yi *Biomaterials* **2013**, 34, 10199-208.

[5] X. Ma, Y. Gao, D. Zhao, W. Zhang, W. Zhao, M. Wu, Y. Cui, Q. Li, Z. Zhang,C. Ma *Nanomaterials (Basel)* **2021**, 12,
